# Interacting Quantum Atoms and Multipolar Electrostatic Study of XH···π Interactions

**DOI:** 10.1021/acsomega.3c04149

**Published:** 2023-09-14

**Authors:** Lena Triestram, Fabio Falcioni, Paul L. A. Popelier

**Affiliations:** Department of Chemistry, University of Manchester, Manchester M13 9PL, Great Britain

## Abstract

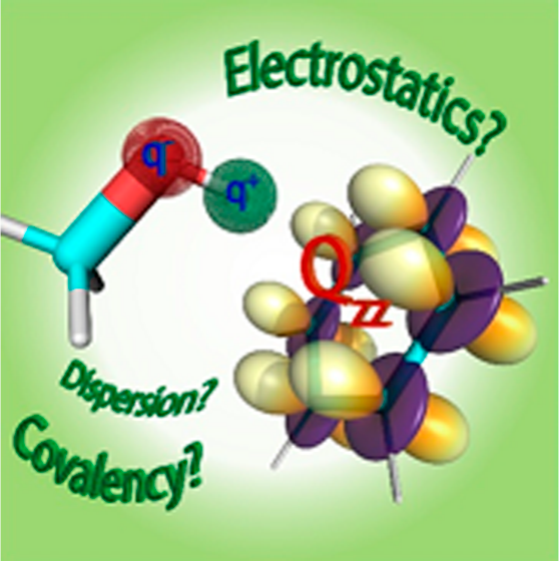

The interaction energies of nine XH···π (X = C, N, and O) benzene-containing van der Waals complexes were analyzed, at the atomic and fragment levels, using QTAIM multipolar electrostatics and the energy partitioning method interacting quantum atoms/fragment (IQA/IQF). These descriptors were paired with the relative energy gradient method, which solidifies the connection between quantum mechanical properties and chemical interpretation. This combination provides a precise understanding, both qualitative and quantitative, of the nature of these interactions, which are ubiquitous in biochemical systems. The formation of the OH···π and NH···π systems is electrostatically driven, with the *Q*_*zz*_ component of the quadrupole moment of the benzene carbons interacting with the charges of X and H in XH. There is the unexpectedly intramonomeric role of X–H (X = O, N) where its electrostatic energy helps the formation of the complex and its covalent energy thwarts it. However, the CH···π interaction is governed by exchange–correlation energies, thereby establishing a covalent character, as opposed to the literature’s designation as a noncovalent interaction. Moreover, dispersion energy is relevant, statically and in absolute terms, but less relevant compared to other energy components in terms of the formation of the complex. Multipolar electrostatics are similar across all systems.

## 1 Introduction

Noncovalent interactions are ubiquitous in chemistry, from enzyme–substrate binding, molecular recognition, and specificity to protein folding and supramolecular chemistry.^1–3^ Within nonstandard noncovalent interactions, such as π···π, lone pair···π, and ion···π interactions, XH···π interactions stand out as a particular case of weak hydrogen bonds, with the π-system as a hydrogen-bond acceptor rather than a single, typically electronegative atom. In this work, we work with two subcategories of these interactions: (i) one where X is a strongly electronegative atom such as N, O, or F and (ii) one where X is a carbon. Crucially for proteins, the CH···π motif has been shown to frequently occur in protein backbones. CH··· π interactions can play a dominant role in substrate–enzyme binding, protein folding, and overall protein stability.^4–9^

The literature on XH···π interactions describes the role of different energy contributions with respect to the total energy of the system, that is, at the molecular level. Sakaki et al.^10^ showed that CH···π interactions, where π is an aromatic compound, are mainly dispersion-driven. More recently, it has been shown that electrostatics are very important in this context. For example, in XOH···π interactions (X = F, Cl, Br, I) between hypohalous acids and a benzene molecule, dispersion and electrostatic energy contribute equally.^11^ Other work on XH···π interactions composed of a benzene and HX (X = H_2_B, H_3_C, H_2_N, HO, or F) has also been carried out.^12^ The relative importance of the dispersion and electrostatic energy varies depending on the polarity of the X–H bond. When X is less electronegative, with groups such as H_2_B and H_3_C, the interaction is more dispersion-driven, while with OH and F, this interaction is electrostatically driven. Studies of Tsuzuki et al.^13,14^ on the magnitude of CH···π interactions between benzene and model hydrocarbons (such as benzene···ethane, benzene···ethene, or benzene···ethyne) show that dispersion energy is again the most relevant. These studies also show that the energy of interaction increases when the hybridization of the carbon decreases. Similar findings were published by Nishio.^15^

A clear presentation of interactions involving aromatic systems emerges from the literature: better computational tools are needed to understand their origins. While the pictorial Hunter–Sanders model^16^ for π–π stacking presents quadrupole moments of benzene as the main contributor to the effect, this does not mean that they are the cause of the interaction in the first place. Other contributions, such as steric effects, induction, and exchange energies, have been shown to be relevant.^17–19^

The aim of the current work is to dissect XH···π interactions in benzene complexes using the relative energy gradient (REG) method.^20^ REG is an automated method to rank atomic contributions according to their role in explaining the energy profile, and thus behavior, of a total system, in this case, a van der Waals complex. It is fruitful to combine REG with interacting quantum atoms (IQA), which is a minimal, parameter-free energy partitioning scheme based on (quantum) topological atoms.^21,22^ The resulting approach is designated as REG-IQA. Moreover, the multipolar expansion of the interatomic electrostatic energy reveals this energy’s fine structure. We call this method REG-MULTI.^23^ The energies are also analyzed using REG-IQF (F = fragments), which is a straightforward extension of REG-IQA thanks to the additivity of topological atoms. Note that when empirical dispersion corrections (such as the D3 dispersion correction) are employed in connection with density functional theory (DFT) calculations, these can be readily paired with the IQA energy partitioning.^24^ In this case, the REG methodology is simply named REG-IQA-D3 (or REG-IQF-D3).

We analyzed seven systems of the NENCI-2021 database and two extra systems.^25^ These nine systems consist of three groups: three OH···π complexes (benzene···AcOH, benzene···MeOH, and benzene···water), three NH···π complexes (benzene···AcNH_2_, benzene···MeNH_2_, and benzene···NMA), and three CH···π complexes (benzene···ethane, benzene···ethene, and benzene···ethyne), with *N*-methylacetamide (NMA) compactly representing the peptide bond, and Me = methyl and Ac = acetyl. We first focus on OH···π and NH···π interactions, due to their similar behavior, and then on CH···π systems.

Two subsequent analyses show the specific role of each interaction between the monomers: (i) REG-IQA-D3, extended with REG-IQF-D3 by grouping relevant atoms, followed by (ii) REG-MULTI, where multipolar electrostatic energies are ranked and REG values are computed against the total multipolar electrostatic energy. The first analysis yields a global picture of the most important energies for the interaction between the monomers, by comparing the interatomic energies (exchange–correlation, electrostatic, and dispersion) and the intra-atomic energies. The second analysis offers a detailed view of the electrostatic energies using multipolar electrostatics.

**Figure 1:**
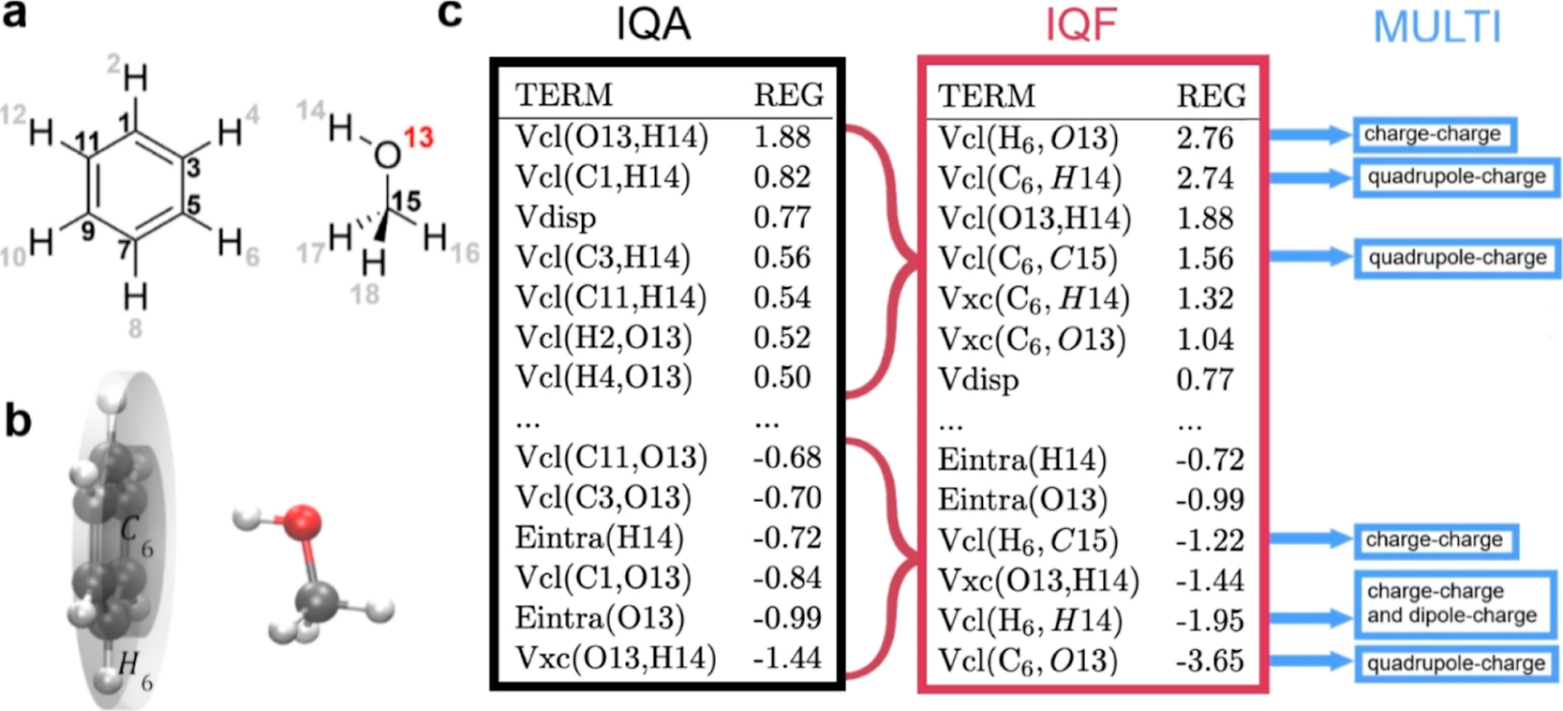
Benzene···methanol complex with (a) the atoms’ numerical labels, (b) the fragments for the REG-IQF groups of benzene, and (c) the all-atom REG-IQA-D3, REG-IQF-D3, and REG-MULTI results. REG values in the IQA table are added following the meaningful subset of all of benzene’s carbon atoms, denoted C_6_ (gray cylinder), and all of benzene’s hydrogen atoms, H_6_ (white cylinder), fragments to obtain the REG-IQF values in the IQF table. The REG-MULTI results (blue) show only the strongest positive and negative contributions to the total electrostatic energy of the corresponding IQF energy term. More detailed REG-MULTI results are given in Table S2.

The benzene···MeOH complex serves as the main example to understand how a REG analysis of OH···π and NH···π systems works. Figure 1c lists the largest positive and negative REG values. Each REG analysis is confined to the energy profile bounded by the complex’s (energy) minimum and a large intermonomeric distance (i.e., “infinity”). IQA energies are additive, and thus, REG-IQA values can be summed to give REG-IQF values. This is also true for REG-IQA-D3 (or REG-IQF-D3) as D3 is an empirical pairwise additive correction. Single atom–atom dispersion REG coefficients are chemically not that meaningful because dispersion is a whole-molecule effect.^26^ Therefore, in this study, *V*_disp_ is a single REG term taken as the sum of all of the chosen pairwise contributions.

## 2 Methods

QTAIM is an established method that portrays atoms as bounded subspaces formed by a bundle of gradient paths (paths of steepest ascent in the electron density) attracted to a nuclear maximum in the electron density.^27^ These atoms are space-filling: they do not overlap or leave gaps between them. They are quantum mechanically well-defined in real space and provide a minimal (i.e., assumption-free) and chemically meaningful partitioning. A straightforward result of the space-filling nature of this partitioning is the additivity of atomic properties, which for our purpose is that of energies
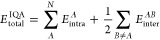
1where the total energy of a system of *N* atoms is partitioned into intra-atomic and interatomic energies. The latter is then divided into chemically meaningful contributions
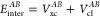
2where V_xc_^*AB*^ is related to covalency, bond order, and hyperconjugation, while *V*_cl_^*AB*^ quantifies the classical electrostatic energy, which governs the effects of charge transfer, polarity, and polarization.^28–32^

Given the general importance of electrostatics in XH···π interactions, this study dissects *V*_cl_^*AB*^ into even more contributions by means of a multipolar expansion truncated at a specific rank. In other words, a series of electrostatic energy terms, expressed in terms of multipole–multipole interactions, make up the total electrostatic contribution
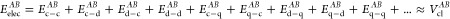
3where c = charge, d = dipole, and q = quadrupole.

This approach is at the heart of long-range electrostatics of the in-house polarizable multipolar force-field FFLUX.^33,34^ In this study, both monomeric multipole moments (i.e., nonpolarized) and dimeric (i.e., polarized) multipole moments were examined. We point out that the term “complex” is more general than “dimer,” which is, strictly speaking, confined to the case where the two monomers are the same molecule. Furthermore, note that the computation of electrostatics through the multipole expansion is computationally much faster than the six-dimensional integration carried out to obtain the exact electrostatic energy, *V*_cl_^*AB*^, at any range.

Another important energy contribution is that of dispersion, which is in principle obtainable within the IQA partitioning through explicit electron correlation.^35^ However, in practice, this involves the handling of a huge two-particle density matrix, too large for the systems in this study when this large (and indeed necessary) number of Gaussian primitives is used. Thus, we opted for a well-known correction, external to IQA but still practically compatible with it, due only to the pairwise additive nature that they have in common. This correction is the D3 dispersion correction, which is readily available in Gaussian 16.^36,37^ Therefore, the total energy between two atoms *A* and *B* becomes
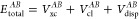
4

The additive and space-filling nature of topological atoms gives the option of summing atomic properties across selected atoms, specifically, functional groups or any meaningful fragment such as a benzene ring or a whole molecule inside a complex. This concept is at the heart of interacting quantum fragments (IQF), which is a generalization of IQA. Due to the π-like nature of the systems analyzed here, it makes sense to analyze certain atoms as a group of a fragment.

Overall, three main quantum-mechanical-based methods are used: IQA, IQF, and MULTI (i.e., multipole moments). These methods are combined with the REG method, which takes as input energies from IQA, IQF, and the in-house molecular dynamics program DL_FFLUX. REG then identifies a small subset of energies that are most relevant to understanding the total energy profile. Note that REG cannot act on a single snapshot of a system but, instead, needs a *change* in energy as a result of a *change* in geometry. Such a change is regulated by the so-called *control coordinate*. If, for example, REG aims to explain a torsional energy barrier, then the control coordinate is the relevant dihedral angle. Note that this coordinate can be thought of as a collective variable in the context of enhanced sampling of molecular dynamics.

REG has been extensively used in recent years to understand various phenomena such as the formation of a water dimer, S_N_^2^ reactions, the fluorine gauche effect, and the origin of the torsional barrier of biphenyl, to name a few.^20,30,38,39^ REG can be combined with any energy partitioning method, but here it takes advantage of the clarity that IQA offers. According to its name, REG compares (hence “relative”) the energy gradient of an atom (or a group thereof) with that of the total system. For that purpose, an ordinary least-squares regression is employed to quantify the correlation between the total energy profile (**E**_total_) and each of the *i*-th subset energy terms (**E**_i_) (that make up the total energy)
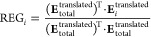
5where
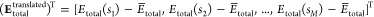

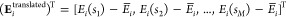
Both the total energy and the *i*-th energy terms are translated over their respective mean, and T marks the transpose. The control coordinate *s* marks the discrete steps taken, from 1 to *M*, which corresponds to a set number of geometries.

The ratio of these gradients is the essential unitless quantity that reveals how important a given subset of energy is. REG ranks these ratios, or so-called REG values, by their magnitude, which corresponds to an importance ranking. In the current case study, the control coordinate is a potential energy surface scan between one monomer and the other monomer in a complex. A REG value can be positive or negative. A positive value means that the specific energy type contributes to the behavior of the total energy, while a negative value means that it goes against it. The analysis is automated through the in-house REG.py library.^40^ More (mathematical) details of the REG method can be found in the original REG paper.^20^ Let us finally emphasize that the sign of a REG value should not be misunderstood. As a matter of caution, for example, it is wrong to think that a negative REG value is always associated with an attractive interaction (and conversely, a positive REG value is associated with a repulsive interaction). Depending on the energy segment analyzed and the energy contribution involved, it may be so that an attractive interatomic interaction ends up with a negative REG value, but this is neither a rule nor a mathematical consequence.

## 3 Computational Details

The initial minimum energy geometries are obtained from the recently published NENCI2021 database.^25^ The geometries of the database have been optimized by the DiStasio group at the MP2/aug-cc-pVTZ level of theory following a specific protocol. However, in this study, it was more practical to employ DFT as a method of choice as implemented in the QTAIM-IQA program AIMAll.^42,43^ Early benchmarks proved the better overall functional to be B3LYP, when paired with the aug-cc-pVTZ basis set, for a good trade-off between accuracy and computational expense (see Section S1 in the Supporting Information).^44^ Note that the NENCI2021 database did not include the benzene···ethane and benzene···methane complexes. These were added manually to the analysis by computing the global energy minimum of the complex at the same MP2/aug-cc-pVTZ level of theory, after generating reasonable geometries in GaussView6.^45^

REG analyses were performed on a potential energy surface scan with the distance between the centroid of the π system (in this case benzene) and the atom of the XH monomer closest to the normal of the benzene plane as the control coordinate. The scan is performed for 15 steps at a step size of 0.2 Å, for a total of 3 Å away from the energy minimum. During the whole scan, the geometries of the constituent monomers were kept frozen at their values in the energy minimum. For each step of the scan, a wave function is obtained for the geometry of the whole complex using the Gaussian 16 package at the B3LYP/aug-cc-pVTZ level of theory with the D3 dispersion correction and Becke–Johnson damping.^36,37,46^ The wave function is then fed into the program AIMAll to obtain IQA energies and the multipole moments of each atom for use in the REG-MULTI analysis. The multipolar electrostatic energies are computed with the in-house program DL-FFLUX, and then passed on to REG.^47^

## 4 Results and Discussion

We analyze interactions via groups of atoms instead of via atom–atom REG-IQA results because the latter may miss the global picture of the intermonomeric interaction. Of course, grouping of interactions is arbitrary, but it is relevant in the context of symmetrical molecules such as benzene. Note that grouping all of the atoms of benzene would defeat the object because this action avoids any atomistic detail. Hence, fragmenting into six carbons and six hydrogens, for example, can be a good compromise between (redundant) atom–atom information and no atomistic detail at all.

An example of the influence of atom grouping (or not) is that of the most important REG-IQA value (Figure 1c), which is the *intra*molecular interaction *V*_cl_(O13, H14). Here, two oppositely charged atoms interact and their negative electrostatic energy must become more negative because their interaction stabilizes the complex’s formation. Because the monomeric geometries are frozen, this effect is only possible by increasing polarization due to the benzene. Indeed, O13 becomes 0.019 *e* more negative (from “infinity” to the minimum), while H14 becomes more positive by 0.003 *e*. Yet, grouping the atoms, as in REG-IQF (Figure 1c), shows the reduced importance of the intramolecular *V*_cl_(O13, H14) compared to the more intuitive intermonomeric interactions.

The four most stabilizing (i.e., positive) REG-IQF values are all electrostatic, with *V*_cl_(H_6_, O13) being the most important, between the group of six hydrogens in benzene and oxygen. REG-MULTI shows that this interaction is dominated by the charge–charge (Figure 1c) contribution. Its energy is negative due to the positive charges on the hydrogens (+0.02 *e*) and the negative charge on oxygen. Hence, the energy becomes more negative (i.e., stabilizing) as the intermonomeric distance shortens. This interaction is closely followed by *V*_cl_(C_6_, H14), between the six carbons in benzene and the hydroxylic hydrogen. This time, the quadrupole moments of the carbons predominately interact with the charge on the hydrogen (Figure 1c). This six-carbon fragment also stabilizes the complex, but then in a weaker fourth place, by interacting with methanol’s carbon [i.e., *V*_cl_(C_6_, C15)].

The next two most important interactions that help the formation of the complex are both of the exchange–correlation type: *V*_xc_(C_6_, H14) and *V*_xc_(C_6_, O13), having C_6_ in common. This interaction only becomes visible as a group interaction (i.e., C_6_) rather than as atomic interactions. Although called “through-space” or “noncovalent,” we emphasize this interaction’s non-negligible covalent character. It is remarkable that this covalent contribution is more important than the dispersion energy of the whole system, *V*_disp_, in the seventh place. However, the latter was the third most positive REG-IQA term before becoming overwhelmed by electrostatic effects when shifting to REG-IQF. This result is consistent with that of another study showing that the dispersion energy is usually more relevant in BH···π and CH···π systems, whereas electrostatic energy dominates for NH···π, OH···π, and FH···π interactions.^12^

Negative REG values represent interactions that are unfavorable to the complex’s formation. Here, electrostatic effects play an important role (Figure 1c), especially the dominant electrostatic repulsion between O and the benzene ring, *V*_cl_(C_6_, O13). However, the electrostatic fine structure reveals that quadrupole(on C)–charge(on O) governs. Furthermore, some unfavorable steric repulsion is caused by the O–H group, expressed by an increase in the intra-atomic energies *E*_intra_(O13) and *E*_intra_(H14).

Similar observations are made for all the OH···π and NH···π systems that were analyzed (Section S2). Overall, the largest REG values for both attractive and repulsive contributions come from electrostatic energy. Across all systems, and even after atom grouping, there are two important intramonomeric interactions that involve the XH bond: its electrostatic energy [*V*_cl_(X, H) in the third, fourth, or fifth place in REG-IQF] helps the formation of the complex, whereas the exchange–correlation energy [*V*_xc_(X, H) in the second to sixth place] is destabilizing.

Dispersion is an important energy contribution, but it is less important than electrostatics. Hence, the REG-IQF-D3 results, for the multitude of systems, show the need for a full dissection of the role of electrostatics, which is carried out here using REG-MULTI and which highlights the important multipole–multipole interactions. Six carbons (C_6_) and six hydrogens (H_6_) of benzene are grouped for each type of multipolar interaction between those groups and the single atoms of the other monomer. Figure 1c shows that the largest REG values in REG-MULTI are mostly charge–charge and quadrupole–charge interactions between methanol’s OH and the C_6_ or H_6_ groups. This is also the case in all the other OH···π and NH···π complexes (Sections S2.2 to S2.6).

**Figure 2:**
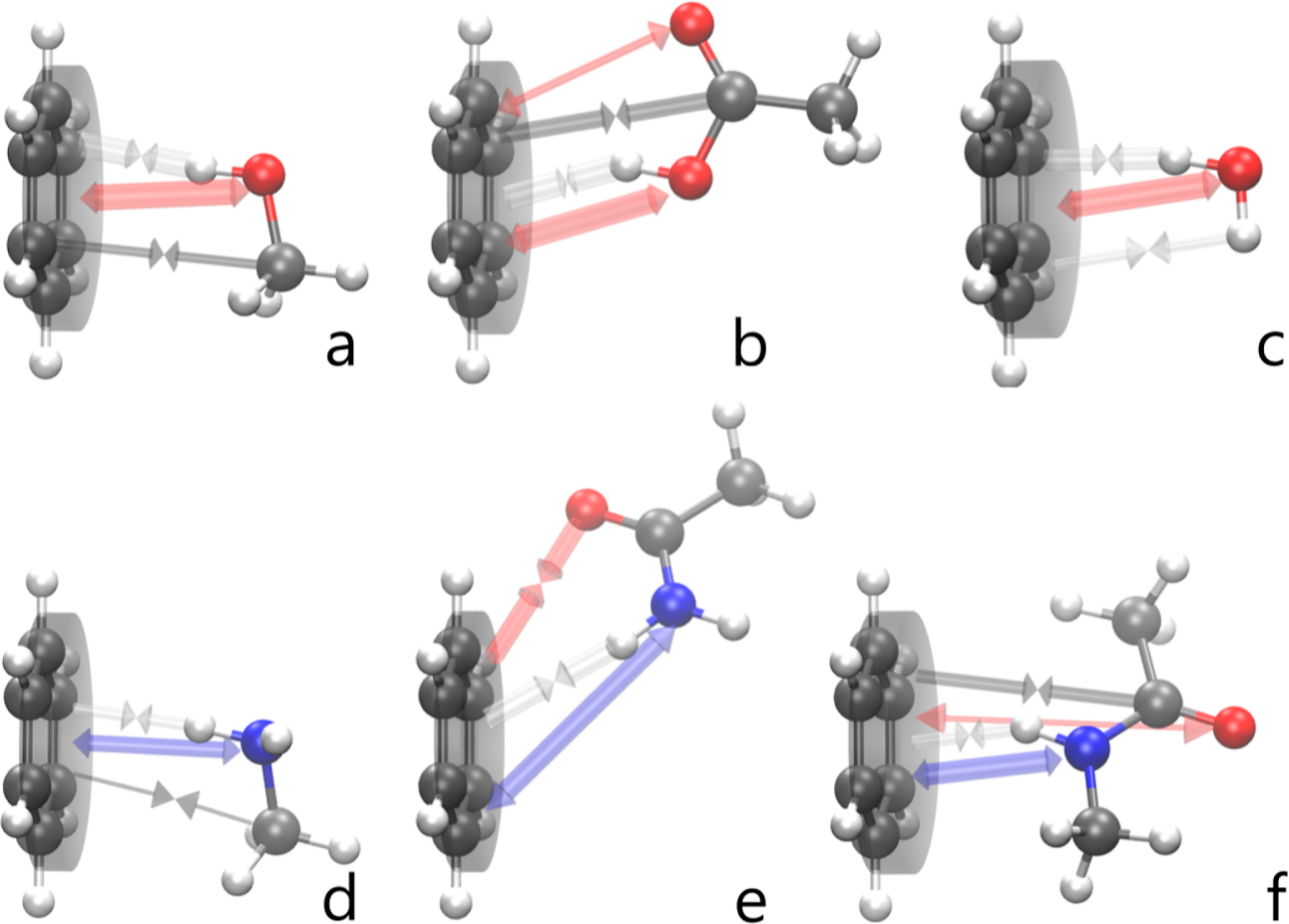
Representation of the most important electrostatic quadrupole–charge interactions based on the REG-MULTI results between the C_6_ fragment (represented by the gray cylinder) and (a) MeOH, (b) AcOH, (c) water, (d) MeNH_2_, (e) AcNH_2_, and (f) NMA (“peptide”). The arrows pointing toward each other represent attractive contributions, while the outwardly pointing arrows represent repulsive contributions. Each arrow has a mixed color (e.g., black and white = gray) representing the two elements interacting (e.g., C = black, H = white). An arrow’s width is proportional to the corresponding REG value. Image generated with VMD.^48^

Figure 2 shows a one-glance representation of the sum of the quadrupole–charge interactions for all six NH···π and OH···π systems. The gray cylinder represents the *Q*_*zz*_ components of C_6_‘s combined atomic quadrupole moments. The attractive intermonomeric energies correspond to C_6_‘s quadrupole moment interacting with the charge of either (i) the H (of the second monomer) pointing toward benzene or (ii) the charge of the C (of the second monomer) closest to benzene’s ring. However, in complexes *c* and *e*, this interaction occurs with oxygen instead of carbon.

The repulsive aspect is mainly driven by quadrupole–charge interactions between the C_6_ fragment and X (X = N, O) of the second monomer. Although there are similar conclusions across the complexes, some results show a strong geometric dependence. For instance, the minimum geometry of AcNH_2_···benzene (*e* in Figure 2) is less centered compared to other complexes because AcNH_2_ mostly interacts with only a small part of benzene. Indeed, O interacts attractively with the side of the benzene ring. In this complex, there are no relevant quadrupole–charge–repellent interactions between C_6_ and the carbons of AcNH_2_. Thus, this NH···π interaction is driven by both H and O of the monomer interacting with the quadrupole moments of benzene’s carbons, with repulsion coming from the N atom. The water···benzene complex also behaves slightly differently from the other complexes. Both hydrogens interact with the C_6_ fragment quadrupole moments. As expected, the interaction of the hydrogen lying directly on top of the benzene (i.e., the H in XH···π) interacts more strongly than the other one because, being more centered, it interacts with *all* of benzene’s carbons.

In summary, REG-MULTI shows that there is a general trend in the electrostatics of NH···π and OH···π interactions across systems. Quadrupole moments of the benzene carbons attract the H atom but repel the N or O atom by interacting with their net atomic charge. Moreover, an attraction is also observed to the α-carbon (directly attached to X) due to its positive charge caused by the electronegative X atom.

On the other side of the spectrum, CH···π are usually classified as XH···π interactions, but we show here that their behavior is very different compared to that of previous systems.^12^ Indeed, the former are mostly driven by dispersion and exchange–correlation energies. REG-IQF-D3 results show that dispersion energy takes up a larger part in CH···π systems than in OH···π and NH···π systems, which is consistent with a previous study and chemical intuition.^12^ For the benzene···ethane and benzene···ethene complexes, the corresponding REG coefficient (*V*_disp_) is the second most important one, but it is less important in the benzene···ethyne complex where it is only the seventh most important (Tables S13–S15).

**Figure 3:**
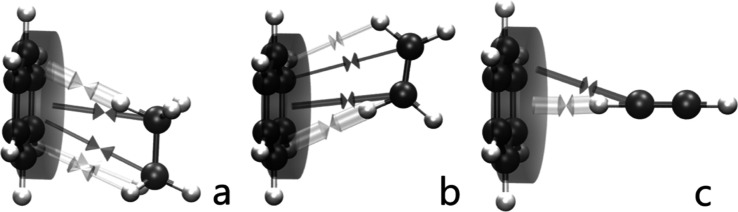
Representation of the most important intermonomeric exchange–correlation interactions between the C_6_ fragment and (a) ethane, (b) ethane, and (c) ethyne, based on the REG-IQF results. The same arrow conventions as in Figure 2.

Figure 3 shows the REG-IQF-D3 results for CH···π complexes. The carbon in the *X* position is less electronegative than the previous X atoms. This carbon cannot change the electron density of its surrounding atoms enough to generate a strong electrostatic attraction with the benzene. The dispersion energy is the largest (by absolute value) energy contribution for all three CH···π complexes (between −66 and −79 kJ/mol at their minimum geometries, as shown in Figure 3), but it does not have the largest REG value. For example, in the benzene···ethane and benzene···ethene complexes, the dispersion’s REG value is half as important as that of the exchange–correlation energy between C_6_ and H in CH (Tables S13 and S14). This is due to the dynamical nature of the REG-IQA-D3 analysis, which inspects the *formation* of the CH···π interaction rather than just the single-point energy minimum. In other words, the dispersion energy is large, but it only serves as a “background” that changes relatively slowly with geometry, compared to the exchange–correlation energy between the benzene carbons and the hydrogen (of the other monomer) closest to the ring [i.e., *V*_xc_(C_6_, H14)].

The largest (by absolute value) exchange–correlation energy [*V*_xc_(C_6_, H14)] between the complexes at their energy minimum geometry is ∼−30 kJ/mol in the CH···π complexes. Although the exchange–correlation energy already appears among the most important REG-IQF values in OH···π and NH···π systems, it plays a larger role in CH···π interactions. Indeed, the total exchange–correlation energy varies by ∼35 kJ/mol between the geometry of the energy minimum and that at maximum monomer separation, while the dispersion energy varies by ∼10 kJ/mol in the CH···π systems. Figure 3 shows two types of exchange–correlation energies: (i) between C_6_ and the carbons of the second monomer and (ii) between C_6_ and the hydrogens of the second monomer. Although the literature reports CH···π interactions as “noncovalent interactions,” REG-IQF concludes through-space exchange–correlation, which is connected to covalency.^12,49–51^ This finding ascribes an unusually covalent character to what is deemed to be a dispersion-driven interaction.

Moving from the covalent to the electrostatic picture for CH···π complexes, the literature’s view that electrostatics are not relevant for CH···π interactions is not true for benzene···ethyne. Here, the sp carbons are more electronegative and thus make the hydrogens electrostatically more relevant, as is clear from Table S15. One of the most important positive multipolar electrostatic interactions in benzene···ethyne is the quadrupole–quadrupole (q–q) interaction between C_6_ and an ethyne carbon (C13 in Figure S41). However, REG-IQF does not list *V*_cl_(C_6_, C13) as an important REG value (Table S15). This is because *V*_cl_ is, in principle, a sum of all possible multipolar contributions, both positive and negative, which balance each other out, while REG-MULTI presents them in detail. The main difference between benzene···ethyne and the other XH···π systems, in terms of electrostatics, is the strong contribution of the quadrupole moments of its carbon atoms. Indeed, quadrupole moments of ethyne’s carbons interact with the quadrupole moments of benzene, overcoming the C_6_–C quadrupole–charge repulsive interaction (Table S16).

## 5 Conclusions

In summary, a common outcome is clear across the three types of XH···π systems: the quadrupole moments of benzene’s carbons, specifically *Q*_*zz*_, which lies along an axis perpendicular to the benzene ring, play a prominent electrostatic role in these interactions. This idea goes back to the early 1990s when Hunter and Sanders aimed to understand the formation of π···π stacking interaction by “mimicking” point charges in different positions around benzene carbons, reaching a qualitative understanding of the π···π concept.^16^ Given the physically sound IQA method, we hereby confirm and quantitatively improve their results, albeit on XH···π interactions. Moreover, it is clear from the dynamical study of REG-MULTI that quadrupole moments of the benzene can be connected to the textbook concept of “π” interactions. However, no orbitals are directly involved in the context of REG-IQF or REG-MULTI because QTAIM, and thus IQA, is an orbital-invariant method.^21,52^ Incidentally, one can argue that it is actually incorrect to give an orbital-based name (π) to an interaction that can be explained without referring to orbitals, as we do here. More results on the interactions between aromatic compounds and ions seem to point in the same direction: strong quadrupole–charge interactions are reported by several studies, where the strength of the electrostatic interactions in those systems plays an essential role in the enantioselectivity.^53–55^ Moreover, the quadrupolar electrostatics of carbon atoms of aromatic rings have been demonstrated to be proper descriptors of aromatic electrophilic substitution reactions, thereby strengthening the importance of the development of methods to understand electrostatic effects in such context.^56,57^

Furthermore, two major points follow from the REG-IQF-D3 analysis. The first is the intramonomeric role of X–H (X = O, N), where its electrostatic energy helps the formation of the complex and its covalent energy thwarts it. The second is that there is a non-negligible through-space covalent component to the XH···π interaction, coming from an exchange–correlation contribution. Thus, the textbook term “noncovalent” is too extreme and potentially misleading because we find that it can be more important than dispersion energy.

Overall, this makes the REG-IQF-D3 and REG-MULTI interesting tools to understand and classify interactions involving aromatic systems purely by employing a physically sound picture of energy components originating from a full dissection of molecular energies at the atomistic level.

## Supporting Information Available

NENCI2021 database partial benchmarks; and monomeric versus dimeric wave functions: MeOH···benzene complex, AcNH_2_···benzene complex, AcOH···benzene complex, MeNH_2_···benzene complex, NMA···benzene complex, water···benzene complex, ethane···benzene complex, ethene···benzene complex, and ethyne···benzene complex (PDF)
