# Supplementary material for: Interacting Quantum Atoms and Multipolar Electrostatic Study of XH···π Interactions
Source: ACS Omega. 2023 Sep 14;8(38):34844–51. doi: 10.1021/acsomega.3c04149 (PMC10535255; doi:10.1021/acsomega.3c04149)
Supplement: Supplementary file 1 — ao3c04149_si_001.pdf [file ao3c04149_si_001.pdf]

Supporting Information

An Interacting Quantum Atoms and Multipolar Electrostatics Study of XH…π Interactions

Lena Triestram, Fabio Falcioni and Paul L.A. Popelier*

Department of Chemistry, University of Manchester, Manchester M13 9PL, Great Britain

*E-mail: pla@manchester.ac.uk

Contents

Section S1. NENCI2021 Database Partial Benchmarks 3

Section S2. Monomeric *versus* Dimeric wave function 10

S2.1 MeOH…benzene complex 11

S2.2 $AcNH_2$…benzene complex 16

S2.3 AcOH…benzene complex 21

S2.4 $MeNH_2$…benzene complex 26

S2.5 NMA…benzene complex 31

S2.6 Water…benzene complex 36

S2.7 Ethane…benzene complex 41

S2.8 Ethene…benzene complex 43

S2.9 Ethyne…benzene complex 45

Section S1. NENCI2021 Database Partial Benchmarks

This study involves the use of relevant geometries that probe XH…π interactions. These are taken from the NENCI2021 database. The paper[15] associated with this database presents and gives access to high-level (e.g. CCSD(T)/CBS) single-point energies across potential energy surfaces of many different non-covalent complexes computed at the MP2/aug-cc-pVTZ level of theory. However, the paper does not show benchmarks of post-HF, Density Functional Theory (DFT) or other Quantum Mechanics (QM) methods that are relevant for our purpose. DFT was more suitable for our study because the program AIMAll[16], which was used for the IQA analysis, is not capable of obtaining two-electron properties from post-HF wave functions. However, our in-house program MORFI is capable to do so but the computational cost is currently not justifiable.

After a private communication with DiStasio's group on their upcoming second part of the NENCI2021 Database, which will show thorough benchmarks across all geometries of the database, we still decided to perform some partial benchmarks on a subset of 5 systems relevant to different non-covalent interactions. Specifically, the 5 systems analysed were:

- Water dimer
- Benzene dimer (π…π)
- Benzene...ethyne complex (CH...π)
- Benzene…F^- complex (anion…π)
- Benzene...Li^+ complex (cation...π)

These systems are shown in Figure S1. The main goal was to find a DFT functional that most resembles the shape of the 'gold standard' CCSD(T)/CBS potential energy surface (PES).

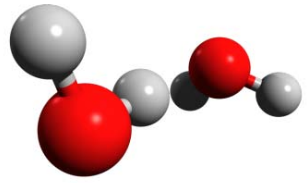

(a) water dimer

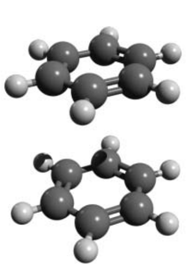

(b) benzene dimer

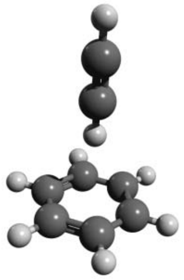

(c) benzene...ethyne complex

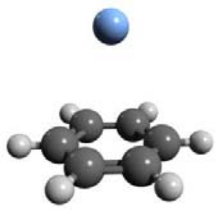

(d) benzene... F^- complex

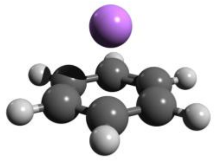

(e) benzene... Li^+ complex

Figure S1. Ball-and-stick representations of the minimum energy geometries of all five benchmark complexes.

The potential energy surfaces of all 5 complexes are shown in Figures S2 to S6, where each figure has a top panel with a full view of the PES and a bottom panel with a blown-up segment of the PES.

Starting from the water dimer in Figure S2 we can clearly see that the pure B3LYP level lacks any dispersion (as expected) and therefore generates an energy profile that substantially deviates from the 'gold' standard CCSD(T)/CBS profile. Note that the latter is henceforth called the reference energy. This conclusion can be drawn for all other systems except the benzene…Li^+ complex.

Given the small size of the water dimer, tests with larger basis sets (aug-cc-pVQZ and aug-cc-pV5Z) were feasible and these do indeed recover the reference energy better. However, the difference to aug-cc-pVTZ is quite systematic and is only about 0.3 kJ/mol, which is almost negligible.

Moving onto the benzene dimer, Figure S3 shows that the absolute energies are generally well recovered from all levels of theory. Specifically, the B3LYP-D3(BJ)[10, 19] has a curvature very similar to the reference PES, while M06-2X (both with and without D3) has a slight upward trend. It seems that there is a systematic and constant error between B3LYP-D3(BJ) and CCSD(T)/CBS, even if M06-2X has absolute energies that are numerically closer to the latter. Note that D3 (which is well-documented in GAUSSIAN's manual) is parametrised to be element-dependent and it is a pairwise-additive scheme. However, D3 does not consider the 'whole-molecule' effect, which makes it inaccurate when many-body dispersion becomes relevant.

Almost equal points can be made for the benzene…ethyne complex shown in Figure S4. Next we look at the benzene…F^- complex in Figure S5 where both B3LYP-D3(BJ) and M06-2X-D3 are struggling at recovering the CCSD(T)/CBS profile. Indeed, the former has a slightly 'late' energy minimum (i.e. more to the right compared to the reference PES in blue) and the latter an 'early' energy minimum. However, B3LYP-D3(BJ) still seems to have a more similar shape, hence gradients, to CCSD(T)/CBS.

Finally, the benzene…Li^+ complex in Figure S6 is interesting because the B3LYP-only calculations are much closer to the gold standard compared to any other calculation with the addition of dispersion correction (or parametrisation of it for M06-2X only). Moreover, it seems as if one line is missing but actually, M06-2X and M06-2X-D3 lie on top of each other, which means that the dispersion correction does not make any difference for this functional in this case. Note that for Li^+ a custom auxiliary basis set denoted aug-cc-pCVTZ was used. This corresponds to aug-cc-pVTZ but with added polarisation functions for the core electrons.

In conclusion, the overall trend is that B3LYP-D3(BJ) generally performs very well in recovering at least the gradients of the CCSD(T)/CBS PES. This is what is needed as REG acts on energy gradients[12] and is thus immune to systematic energy errors (i.e. quasi-parallel energy profiles). Moreover, for the specific study of XH…π interactions, it performs almost exactly as the reference level of theory.

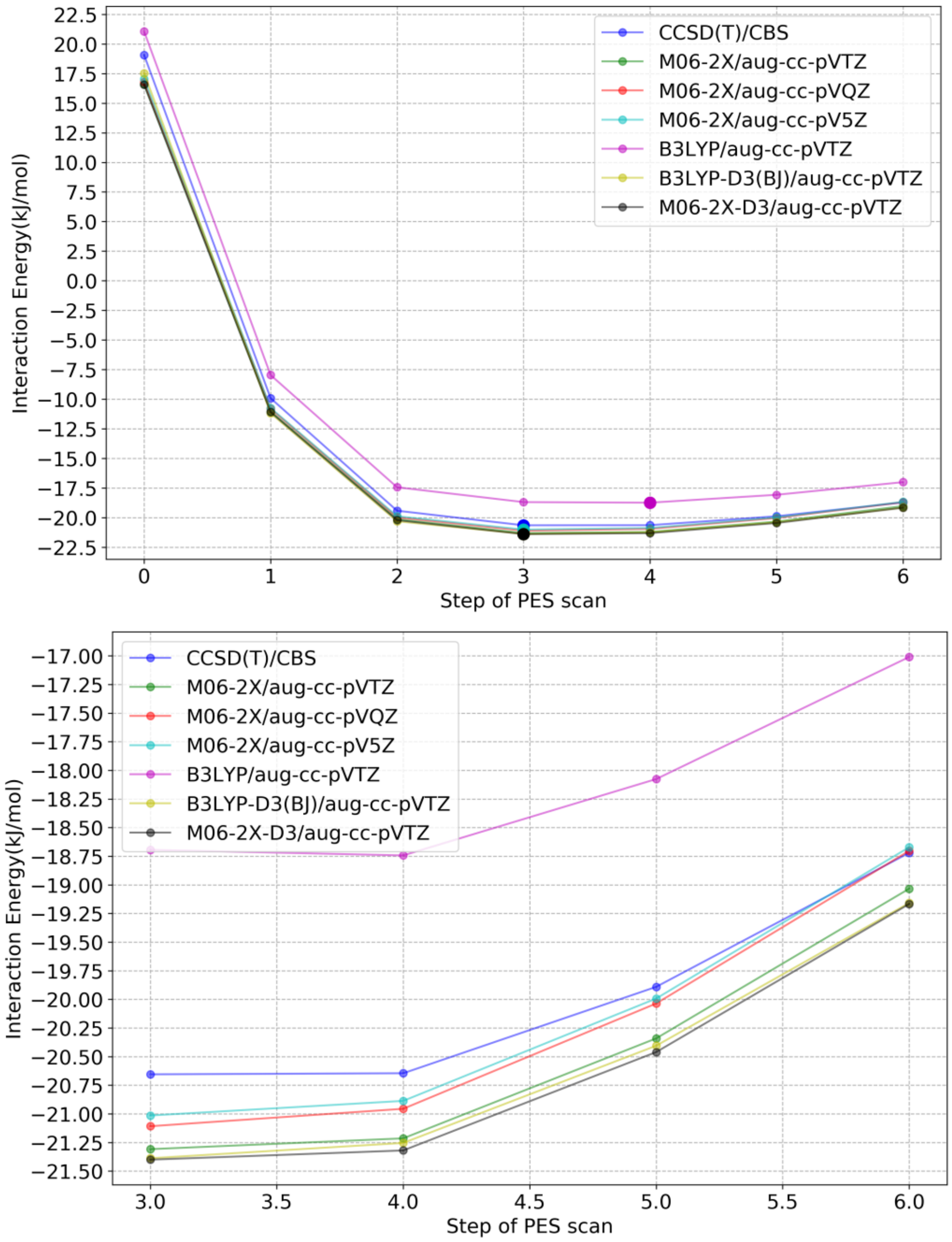

Figure S2. Potential energy surfaces (top=full picture, bottom=zoom in) of the water dimer computed at several levels of theory. Thicker points mark the energy minimum.

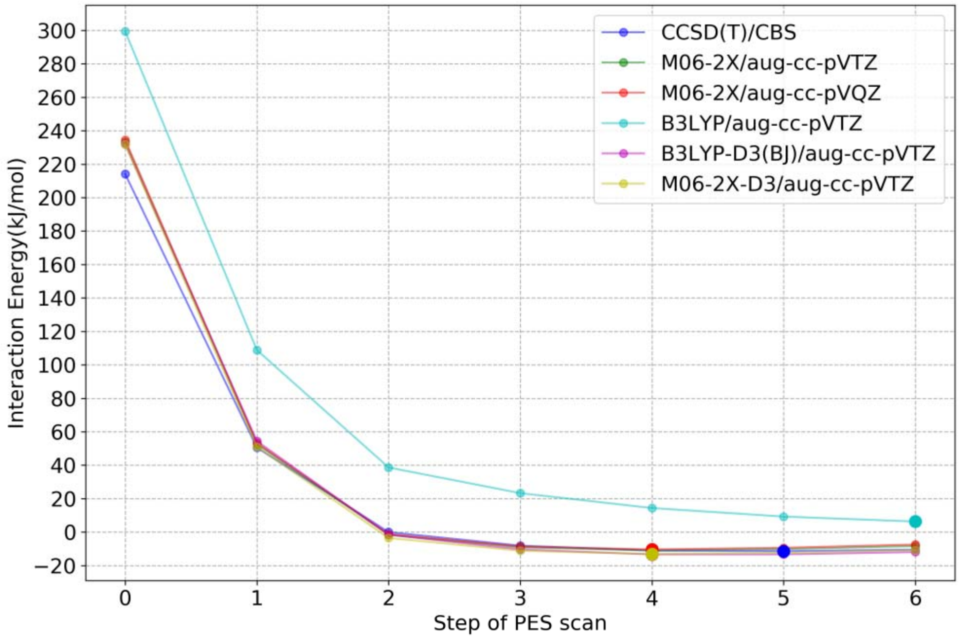

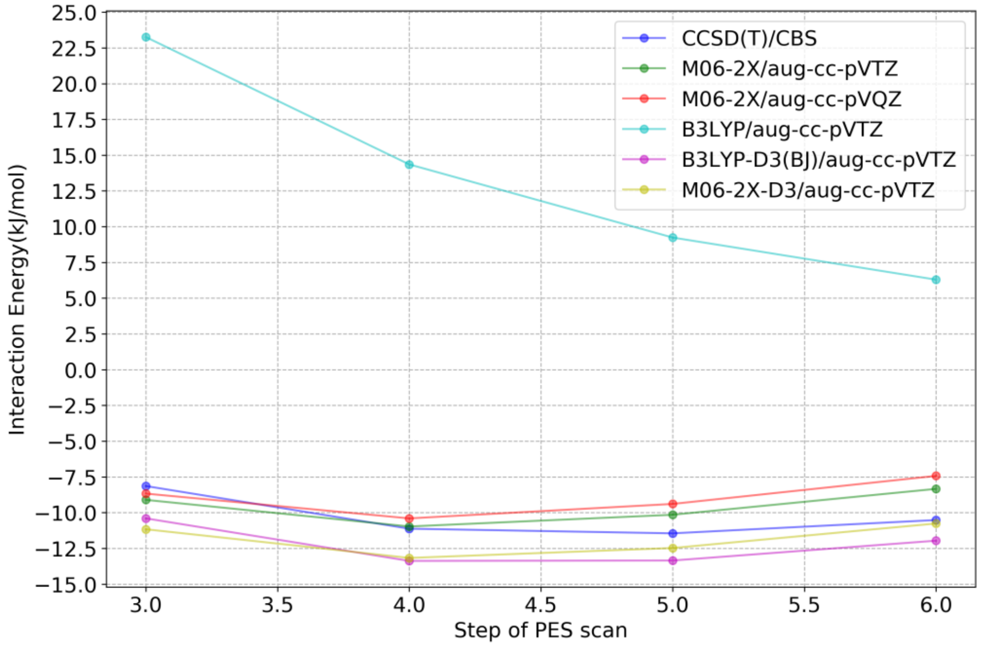

Figure S3. Potential energy surfaces (top=full picture, bottom=zoom in) of the benzene dimer computed at several levels of theory. Thicker points mark the energy minimum.

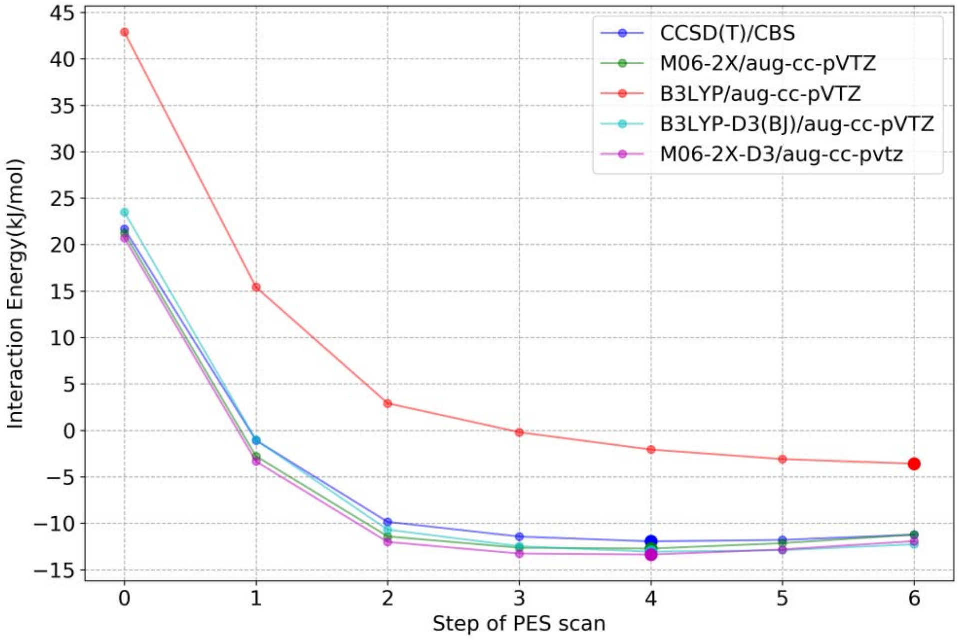

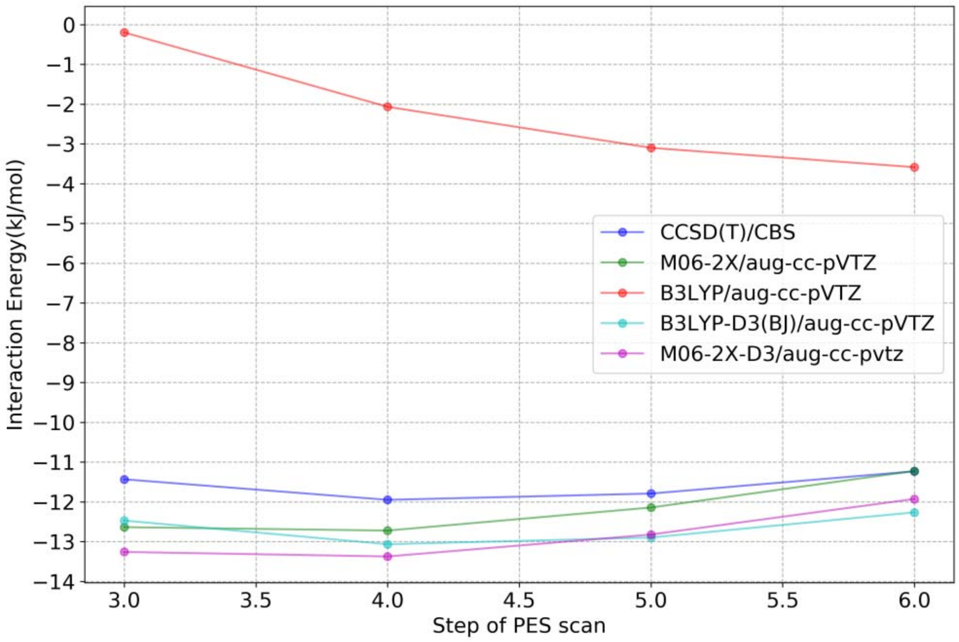

Figure S4. Potential energy surfaces (top=full picture, bottom=zoom in) of the benzene…ethyne complex computed at several levels of theory. Thicker points mark the energy minimum.

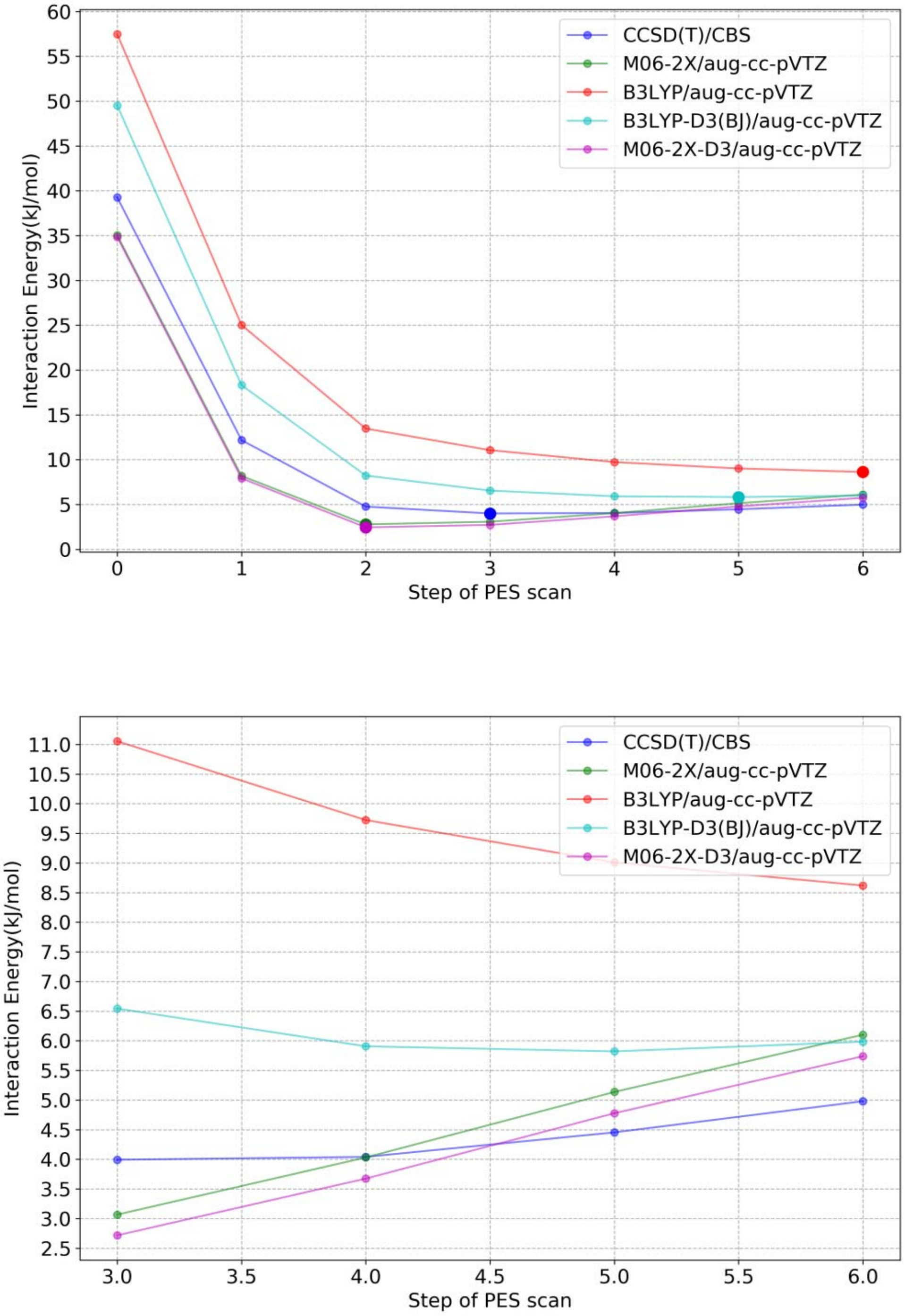

Figure S5. Potential energy surfaces (top=full picture, bottom=zoom in) of the benzene…F^- complex computed at several levels of theory. Thicker points mark the energy minimum.

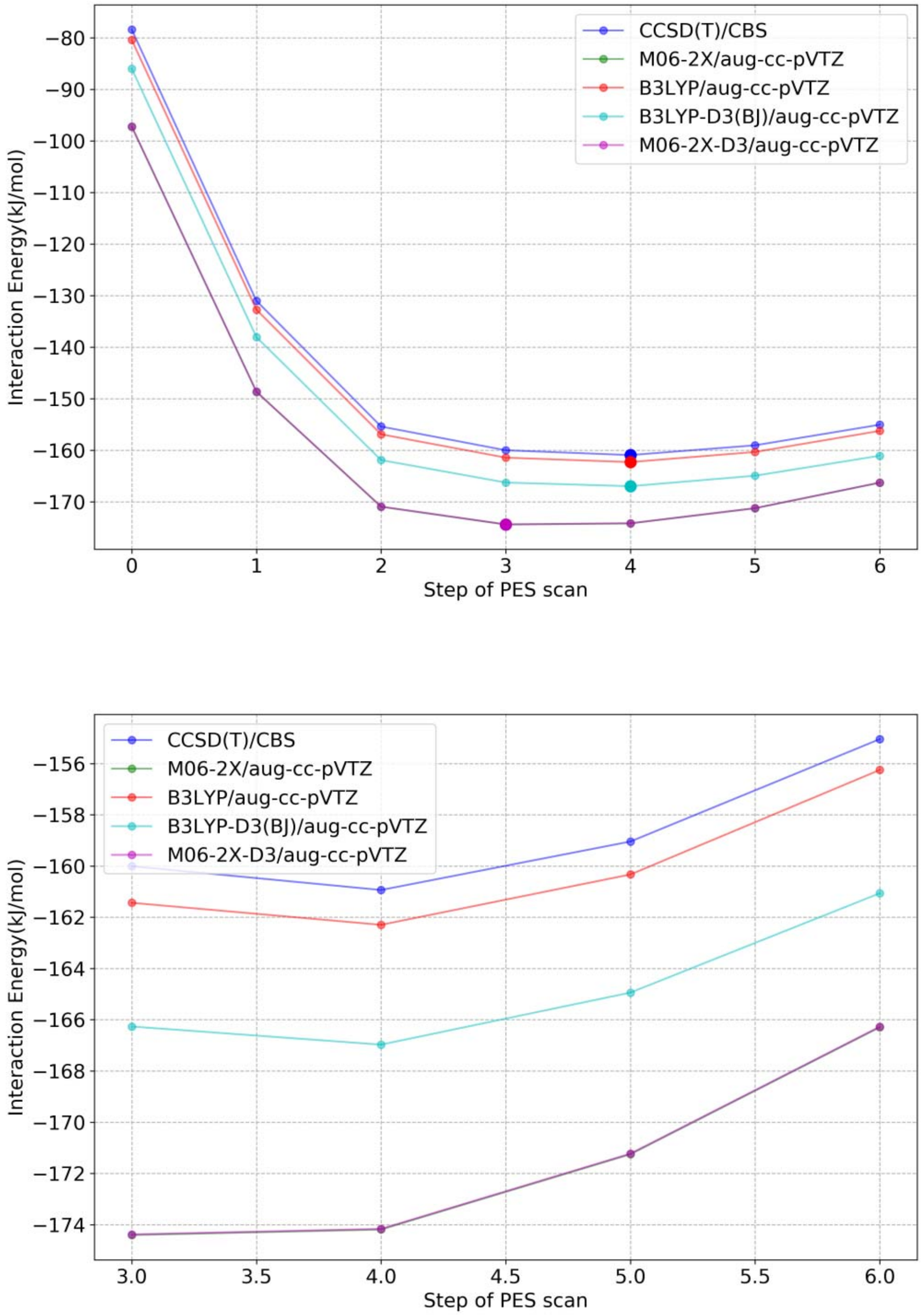

Figure S6. Potential energy surfaces (top=full picture, bottom=zoom in) of the benzene…Li^+ complex computed at several levels of theory. Thicker points mark the energy minimum.

Section S2. Monomeric *versus* Dimeric wave function

The main article presented REG-MULTI results for wave functions of van der Waals complexes, referred to as “dimeric” results for brevity. However, we analysed both monomeric and dimeric results. In the monomeric results, the multipole moments do not change during the analysis because in this method the electron density of one monomer is not affected, quantum mechanically, by the electron density of the other monomer. In other words, the multipole moments calculated with this approach are those calculated as if the monomers were infinitely far apart from each other. Intermolecular polarisation is then omitted but penetration effects occur. In contrast, the dimeric approach is more realistic but also more computationally expensive because the multipole moments are calculated for each step of the PES. The moments change with the change in geometry, and thereby express polarisation effects.

The comparison of the REG results between the monomeric and the dimeric analysis provides insight into intermolecular polarisation effects and their relevance. A REG-MULTI analysis carried out with the monomeric wave function fails to represent the importance of charge/charge interactions. Indeed, charge/charge interactions are going up in the rankings for both positive and negative REG values. More specifically, in the monomeric REG-MULTI they are usually less important than other contributions such as quadrupole-charge interactions, but in the dimeric REG-MULTI they become the most important overall. However, quadrupole/charge interactions are still very important and more so when the quadrupole moments are summed over the benzene carbons (i.e. when we consider a whole-molecule electrostatic effect). In the dimeric analysis, quadrupole/charge rankings generally do not change compared to those associated with monomeric wave functions. This could indicate that there is not much intermolecular polarisation in these systems. Hence, the monomeric wave functions represent well the electrostatic environment of the true (dimeric) complex.

The following subsections (S2.n, n=1,9) show the full results for each of the 9 complexes studied. The information for each complex consists of a number of figures and tables, making up 6 item types per complex, as listed just below. The benzene…methanol complex (Section 2.1) serves as a guide to illustrate this information with specific reference to figure and table numbers. The 6 item types are:

(1) Figure S7 shows the minimum energy geometry and the numerical labels of all atoms.
(2) Figure S8 shows the potential energy surfaces for the REG-IQA analysis.
(3) Table S1 shows the REG values for the REG-IQF analysis.
(4) Table S2 shows the REG values of the REG-MULTI analysis. The IQA terms are marked with colours corresponding to the familiar elemental colours (black=C, white=H, blue=N and red=O), with atom-atom interactions as combined colours, e.g. C…H is grey, which is black combined with white. The notation C_6 refers to the ring of six carbons in benzene and should not be confused with a dispersion coefficient.
(5) Figures S9 and S10 show the convergence graphs of the multipole expansion at a specific rank for selected short-range atom-atom interactions (mainly between a benzene carbon and atom X and H of the corresponding XH…π interaction).
(6) Figure S11 shows a histogram of the change in atomic charge in going from the monomer to the dimer (“complex” in general) in the geometry of the global energy minimum. Note that a positive difference correspondence to a larger net atomic charge in the dimer.

Note that a rank of L’ = 4 means computation of interactions up to hexadecapole-hexadecapole while L’ = 2 is up to quadrupole-quadrupole.

S2.1 MeOH…benzene complex

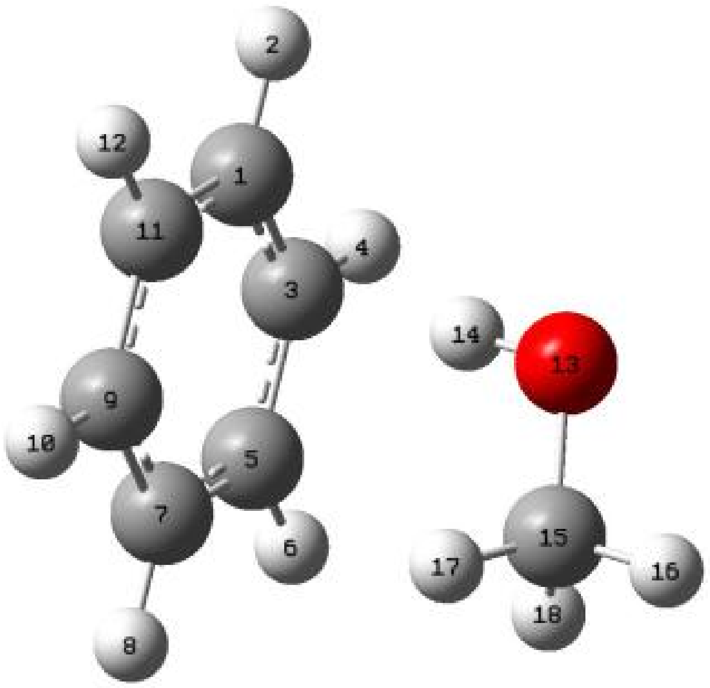

Figure S7. The geometry of the MeOH…benzene complex's energy minimum and its atom labelling.

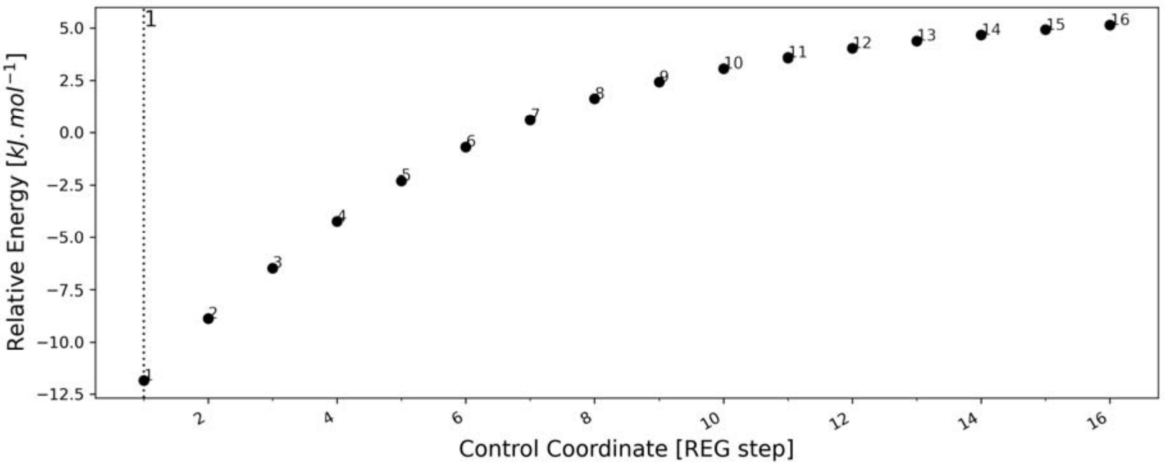

Figure S8. PES of the MeOH…benzene complex. Total wavefunction energy curve considered in the REG-IQA analysis. The Y-axis corresponds to energy relative to the average value over all energies, while the X-axis shows the control coordinate steps starting from the complex minimum (dotted vertical line).

Table S1. Largest positive and negative REG values for the REG-IQF analysis of the MeOH…benzene complex.

TERM	REG
$V_{cl}(H_6,o13)$	2.76
$V_{cl}(C_6,h14)$	2.74
$V_{cl}(o13,h14)$	1.88
$V_{cl}(C_6,c15)$	1.56
$V_{xc}(C_6,h14)$	1.32
$V_{xc}(C_6,o13)$	1.04
V_{disp}	0.77
...	...
$E_{intra}(h14)$	-0.72
$E_{intra}(o13)$	-0.99
$V_{cl}(H_6,c15)$	-1.22
$V_{xc}(o13,h14)$	-1.44
$V_{cl}(H_6,h14)$	-1.95
$V_{cl}(C_6,o13)$	-3.65

Table S2. Largest positive and negative REG values for the MeOH…benzene complex: (left) the monomeric REG-MULTI analysis, (centre) the dimeric REG-MULTI analysis, and (right) the dimeric REG-MULTI analysis modified to show the sum of quadrupole(q)_charge(c) REG values.

TERM	REG	R
c1_h14(q_c)	2.47	0.980
c3_h14(q_c)	1.87	0.988
c11_h14(q_c)	1.82	0.989
c5_h14(q_c)	1.05	0.998
c9_h14(q_c)	1.03	0.998
c7_c15(q_c)	1.00	0.982
c7_h14(q_c)	0.78	0.999
c9_c15(q_c)	0.73	0.992
c5_c15(q_c)	0.72	0.993
.	.	.
c1_o13(q_c)	-1.18	-0.995
c3_o13(q_c)	-1.34	-0.994
c11_o13(q_c)	-1.35	-0.994
c5_o13(q_c)	-1.74	-0.991
c9_o13(q_c)	-1.76	-0.991
c7_o13(q_c)	-1.99	-0.990

TERM	REG	R
c1_h14(q_c)	2.55	0.913
c3_h14(q_c)	1.95	0.931
c11_h14(q_c)	1.88	0.933
c5_h14(q_c)	1.13	0.960
h2_o13(c_c)	1.11	0.933
c9_h14(q_c)	1.09	0.962
h4_o13(c_c)	1.05	0.934
h12_o13(c_c)	1.04	0.935
c7_c15(q_c)	1.01	0.919
c1_h14(c_c)	0.93	0.932
c7_h14(q_c)	0.85	0.974
h6_o13(c_c)	0.80	0.947
h10_o13(c_c)	0.79	0.948
c9_c15(q_c)	0.76	0.942
c5_c15(q_c)	0.75	0.942
.	.	.
c7_o13(q_c)	-1.23	-0.949
c1_o13(c_c)	-1.28	-0.940
c9_o13(q_c)	-1.40	-0.944
c5_o13(q_c)	-1.43	-0.943
c11_o13(q_c)	-1.81	-0.938
c3_o13(q_c)	-1.84	-0.938
c1_o13(q_c)	-2.07	-0.934

TERM	REG
C_6_h14(q_c)	9.46
C_6_c15(q_c)	3.64
h2_o13(c_c)	1.11
h4_o13(c_c)	1.05
h12_o13(c_c)	1.04
c1_h14(c_c)	0.93
h6_o13(c_c)	0.80
h10_o13(c_c)	0.79
.	.
c1_o13(c_c)	-1.28
C_6_o13(q_c)	-9.78

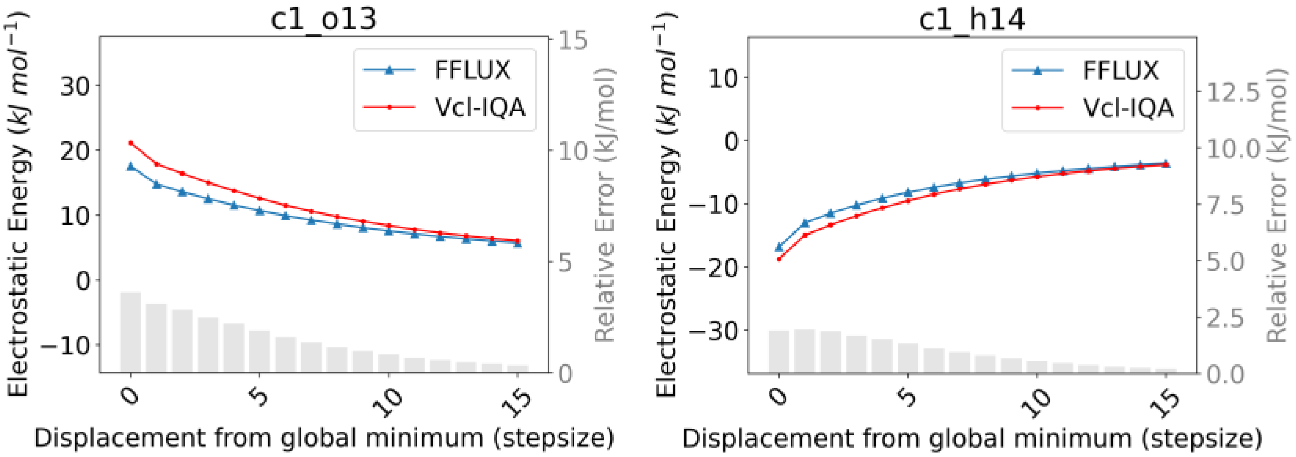

Figure S9. Convergence graphs of the monomeric energies used in REG-MULTI compared to the electrostatic IQA energies for the interactions between the closest atoms in the MeOH…benzene complex. V_{cl} refers to the exact electrostatic energy obtained by 6D integration, while "FFLUX" refers to this energy approximated by multipole expansion at L'=4 truncation, while the grey histogram represents the absolute energy difference. Note that energy electrostatic energy components are only summed up to L=5.

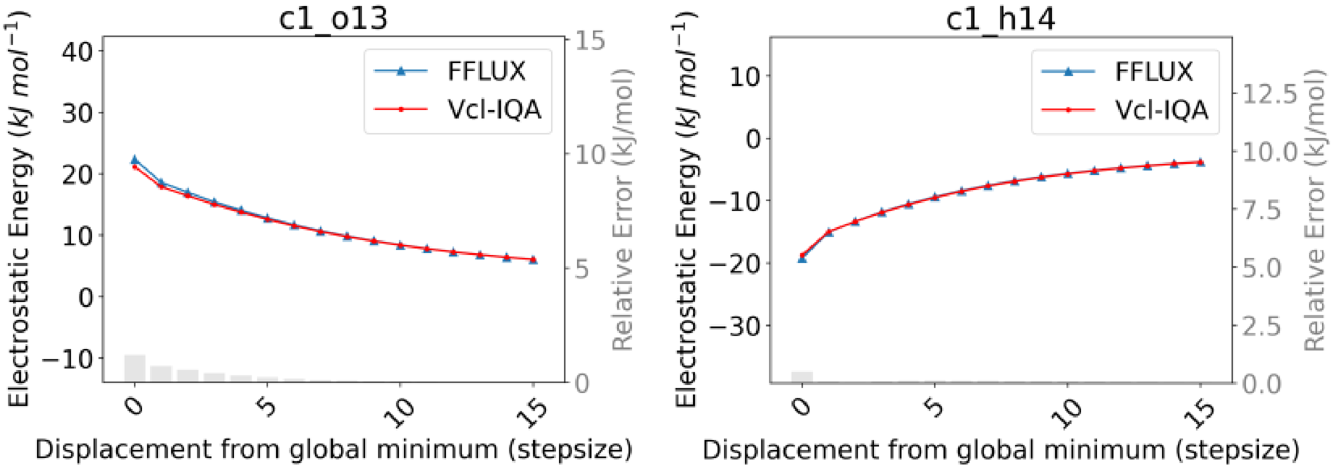

Figure S10. Convergence graphs of the dimeric energies used in REG-MULTI compared to the electrostatic IQA energies for the interactions between the closest atoms in the MeOH…benzene complex. V_{cl} refers to the exact electrostatic energy obtained by 6D integration, while "FFLUX" refers to this energy approximated by multipole expansion at L'=4 truncation, while the grey histogram represents the absolute energy difference. Note that energy electrostatic energy components are only summed up to L=5.

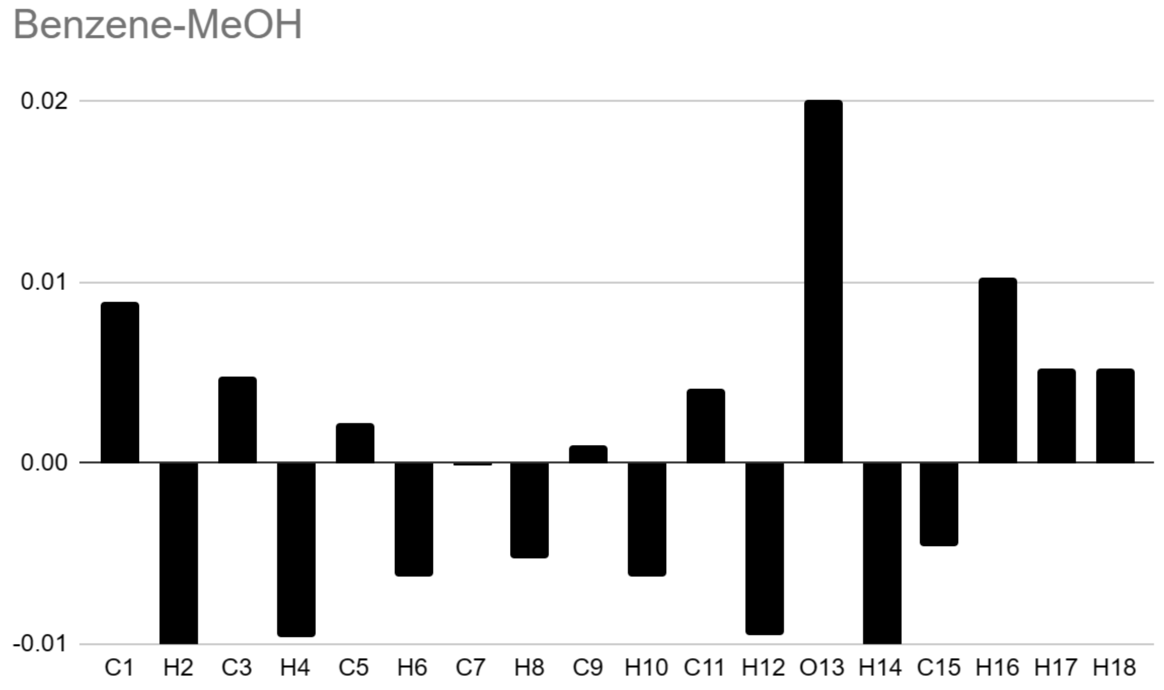

Figure S11. The difference (dimer minus monomer) between the atomic charges (in atomic units of electronic charge, e) of the REG-MULTI analysis (monomeric result) and the atomic charges of the energy minimum's geometry of the REG-MULTI analysis (dimeric result) in the MeOH…benzene complex.

S2.2 AcNH$_2$…benzene complex

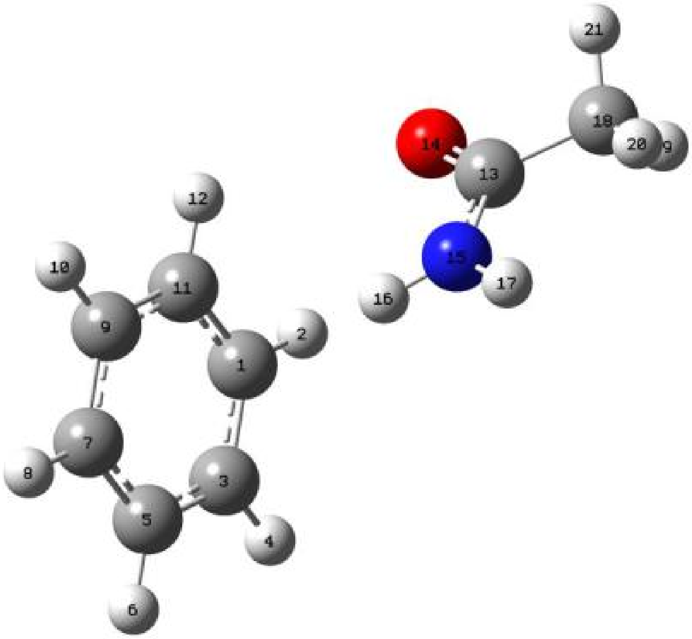

Figure S12. The geometry of the AcNH$_2$…benzene complex's energy minimum and its atom labelling.

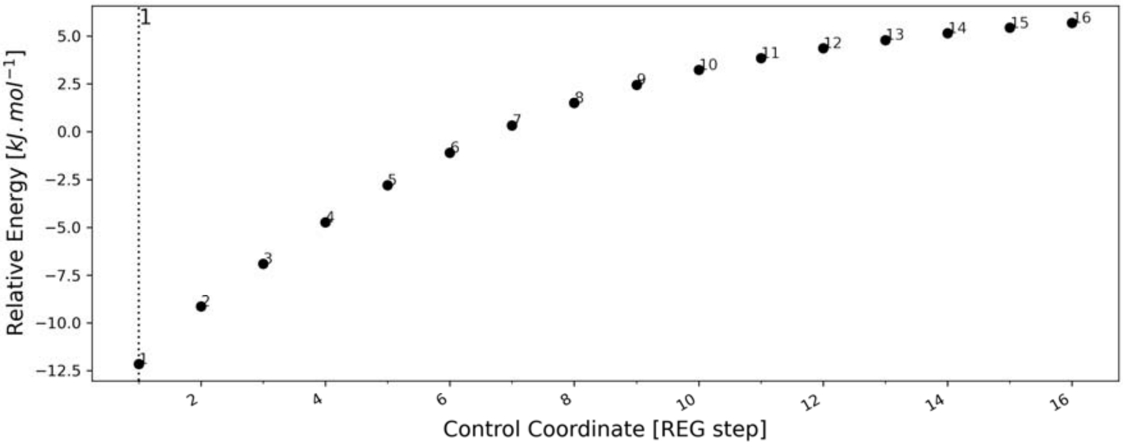

Figure S13. PES of the AcNH$_2$…benzene complex. Total wavefunction energy curve considered in the REG-IQA analysis. The Y-axis corresponds to energy relative to the average value over all energies, while the X-axis shows the control coordinate steps starting from the complex minimum (dotted vertical line).

Table S3. Largest positive and negative REG values of the REG-IQF analysis of the $AcNH_2$…benzene complex.

TERM	REG
$V_{cl}(H_6,n15)$	2.50
$V_{cl}(H_6,o14)$	2.29
$V_{cl}(C_6,c13)$	1.94
$V_{cl}(C_6,h16)$	1.57
$V_{cl}(n15,h16)$	1.25
$V_{xc}(C_6,h16)$	1.02
$V_{cl}(c13,o14)$	0.94
$V_{xc}(H_6,o14)$	0.78
…	…
$E_{intra}(h16)$	-0.74
$V_{xc}(n15,h16)$	-0.99
$V_{cl}(C_6,o14)$	-0.99
$E_{intra}(o14)$	-1.07
$V_{cl}(H_6,h16)$	-1.31
$V_{cl}(C_6,n15)$	-2.59
$V_{cl}(H_6,c13)$	-2.78

Table S4. Largest positive and negative REG values for the $AcNH_2$…benzene complex: (left) the monomeric REG-MULTI analysis, (centre) the dimeric REG-MULTI analysis, and (right) the dimeric REG-MULTI analysis modified to show the sum of quadrupole(q)_charge(c) REG values.

TERM	REG	R
c11_h16(q_c)	1.05	0.998
c1_h16(q_c)	1.03	0.998
c11_o14(q_c)	0.47	0.935
c1_o14(q_c)	0.47	0.932
h12_o14(c_c)	0.39	0.973
h2_o14(c_c)	0.38	0.973
.	.	.
h2_o14(d_c)	-0.50	-0.998
h12_o14(d_c)	-0.50	-0.998
c1_n15(q_c)	-1.05	-0.998
c11_n15(q_c)	-1.05	-0.998

TERM	REG	R
h12_o14(c_c)	1.45	0.999
h2_o14(c_c)	1 .41	0 .999
h12_n15(c_c)	1.20	0.999
h2_n15(c_c)	1.16	0.999
c11_h16(q_c)	0.70	0.998
c1_h16(q_c)	0.69	0.999
c11_c13(c_c)	0.67	0.994
c1_c13(c_c)	0.67	0.993
c1_o14(q_c)	0.38	0.946
c11_o14(q_c)	0.38	0.946
.	.	.
c1_n15(q_c)	-0.67	-0.995
c11_n15(q_c)	-0.67	-0.995
h2_c13(c_c)	-1.41	-0.999
h12_c13(c_c)	-1.44	-0.999

TERM	REG
C_6_h16(q_c)	1.62
C_6_o14(q_c)	1.52
h12_o14(c_c)	1.45
h2_o14(c_c)	1 .41
h12_n15(c_c)	1.20
h2_n15(c_c)	1.16
c11_c13(c_c)	0.67
c1_c13(c_c)	0.67
.	.
h2_c13(c_c)	-1.41
h12_c13(c_c)	-1.44
C_6_n15(q_c)	-1.59

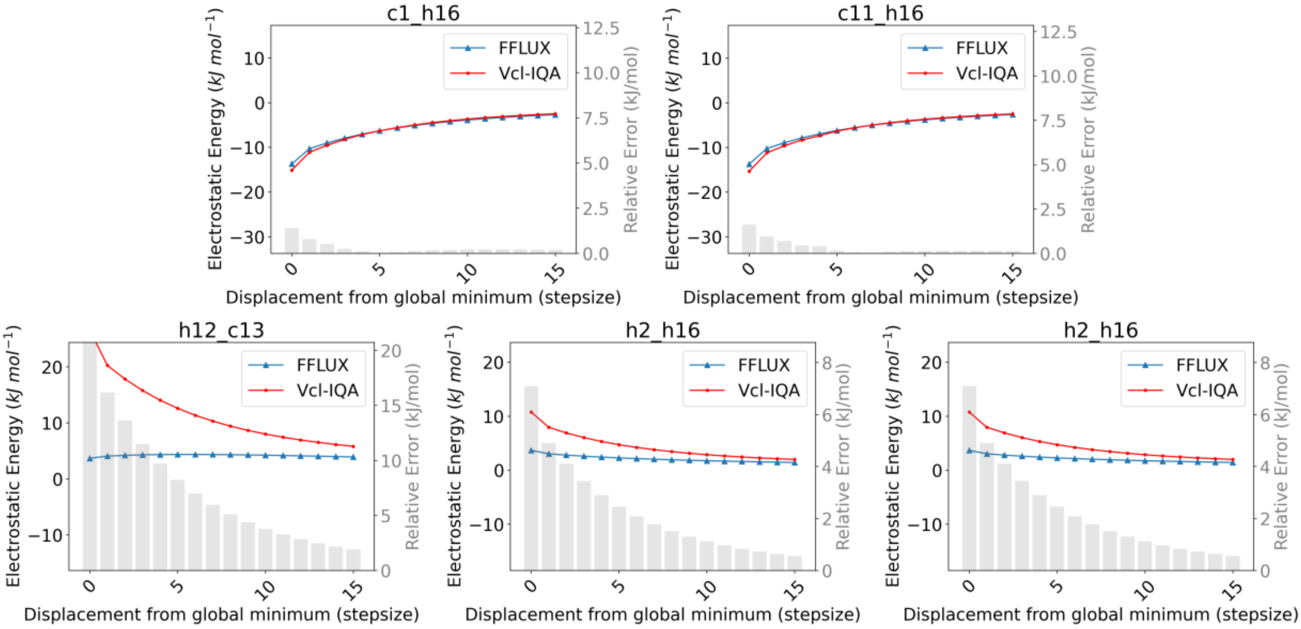

Figure S14. Convergence graphs of the monomeric energies used in REG-MULTI compared to the electrostatic IQA energies for the interactions between the closest atoms in the $AcNH_2$…benzene complex. V_{cl} refers to the exact electrostatic energy obtained by 6D integration, while "FFLUX" refers to this energy approximated by multipole expansion at L'=4 truncation, while the grey histogram represents the absolute energy difference. Note that energy electrostatic energy components are only summed up to L=5.

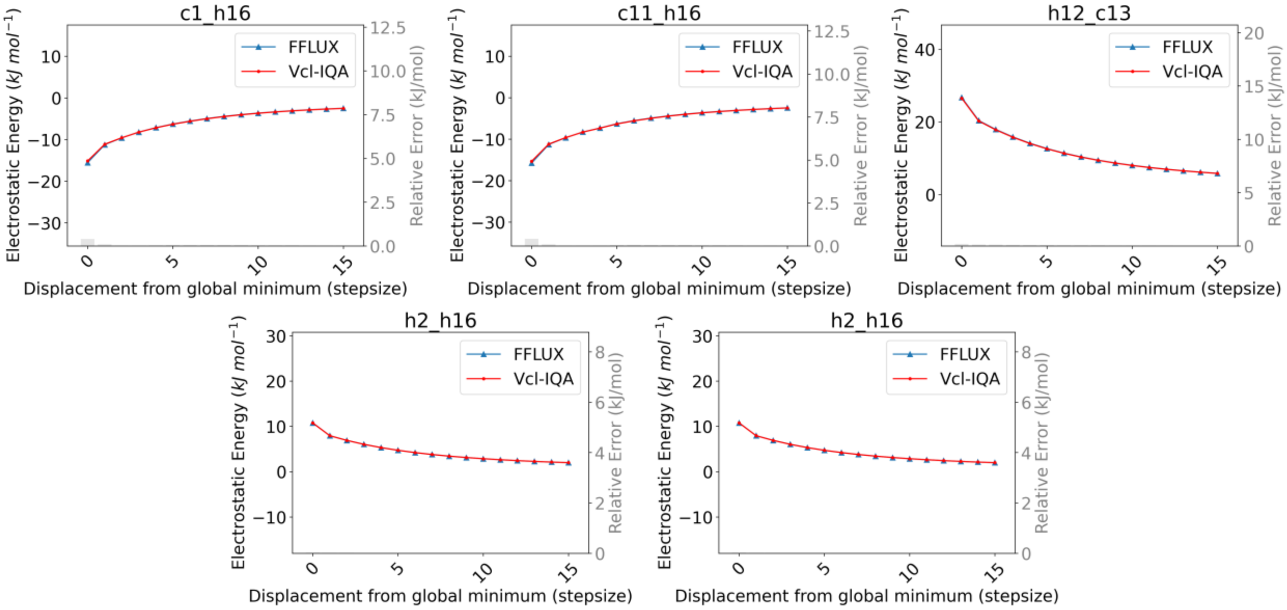

Figure S15. Convergence graphs of the dimeric energies used in REG-MULTI compared to the electrostatic IQA energies for the interactions between the closest atoms in the $AcNH_2$…benzene complex. V_{cl} refers to the exact electrostatic energy obtained by 6D integration, while "FFLUX" refers to this energy approximated by multipole expansion at L'=4 truncation, while the grey histogram represents the absolute energy difference. Note that energy electrostatic energy components are only summed up to L=5.

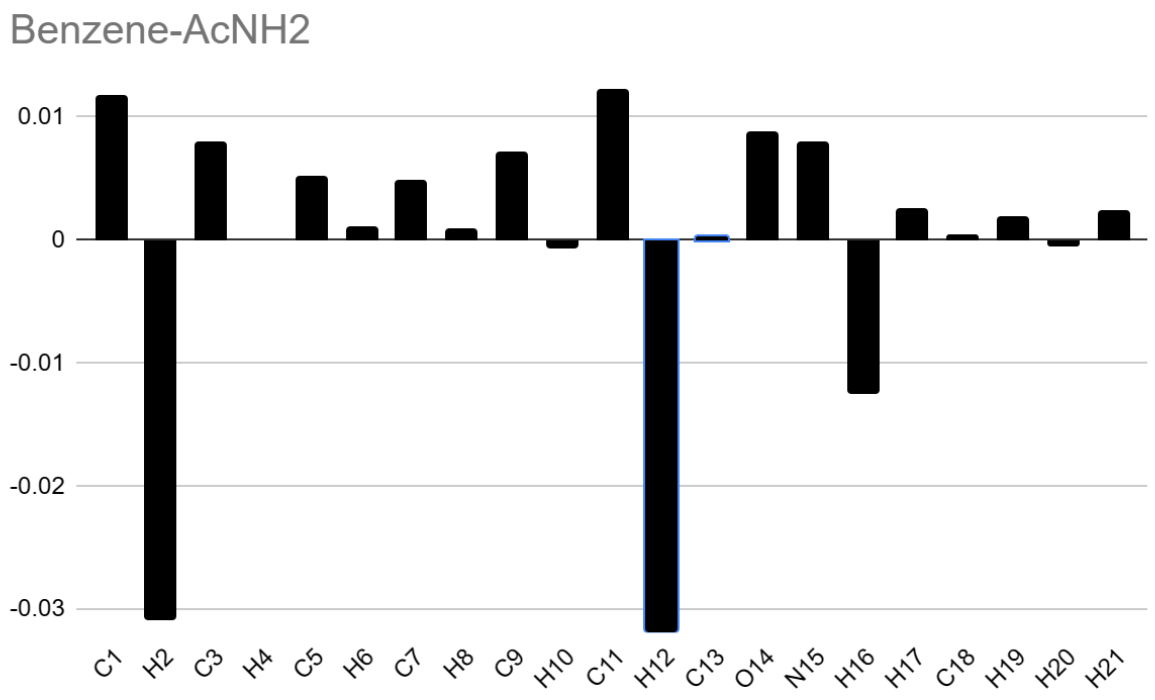

Figure S16. The difference (dimer minus monomer) between the atomic charges (in atomic units of electronic charge, e) of the REG-MULTI analysis (monomeric result) and the atomic charges of the energy minimum's geometry of the REG-MULTI analysis (dimeric result) in the $AcNH_2$…benzene complex.

S2.3 AcOH…benzene complex

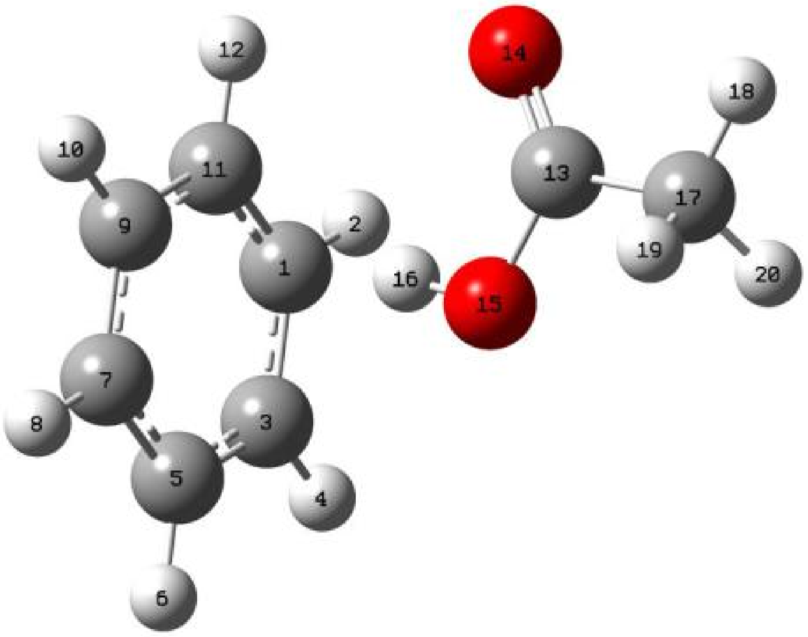

Figure S17. The geometry of the AcOH…benzene complex's energy minimum and its atom labelling.

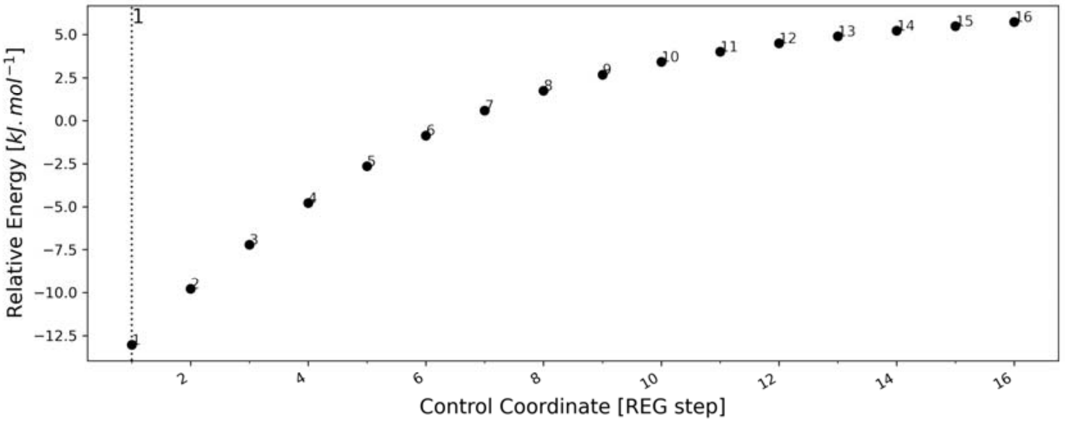

Figure S18. PES of the AcOH…benzene complex. Total wavefunction energy curve considered for the REG-IQA analysis. The Y-axis corresponds to energy relative to the average value over all energies, while the X-axis shows the control coordinate steps starting from the complex minimum (dotted vertical line).

Table S5. Largest positive and negative REG values of the REG-IQF analysis of the AcOH…benzene complex.

**TERM**	**REG**
$V_{cl}(C_6,c13)$	3.57
$V_{cl}(C_6,h16)$	3.04
$V_{cl}(H_6,o15)$	2.88
$V_{cl}(o15,h16)$	1.72
$V_{xc}(C_6,h16)$	1.28
$V_{xc}(C_6,o15)$	1.07
$V_{cl}(c13,o15)$	1.07
…	…
$E_{intra}(o15)$	-1.22
$V_{xc}(o15,h16)$	-1.33
$V_{cl}(H_6,h16)$	-2.16
$V_{cl}(C_6,o14)$	-2.26
$V_{cl}(H_6,c13)$	-3.10
$V_{cl}(C_6,o15)$	-3.79

Table S6. Largest positive and negative REG values for the AcOH…benzene complex: (left) the monomeric REG-MULTI analysis, (centre) the dimeric REG-MULTI analysis, and (right) the dimeric REG-MULTI analysis modified to show the sum of quadrupole(q)_charge(c) REG values.

TERM	REG	R
c11_h16(q_c)	2.50	0.975
c11_c13(q_c)	1.99	0.983
c9_h16(q_c)	1.84	0.986
c1_h16(q_c)	1.72	0.988
c9_c13(q_c)	1.36	0.991
c1_c13(q_c)	1.24	0.992
c7_h16(q_c)	0.99	0.998
c3_h16(q_c)	0.93	0.999
c5_h16(q_c)	0.72	0.998
c7_c13(q_c)	0.62	0.997
c3_c13(q_c)	0.57	0.997
.	.	.
c1_o14(q_c)	-0.60	-0.997
c9_o14(q_c)	-0.73	-0.998
c5_o15(q_c)	-1.17	-0.993
c3_o15(q_c)	-1.28	-0.992
c7_o15(q_c)	-1.31	-0.992
c1_o15(q_c)	-1.58	-0.990
c9_o15(q_c)	-1.63	-0.989
c11_o14(q_c)	-1.71	-0.984
c11_o15(q_c)	-1.81	-0.988

TERM	REG	R
c11_h16(q_c)	2.58	0.955
h12_o14(c_c)	2.21	0.929
c11_c13(q_c)	1.94	0.962
c9_h16(q_c)	1.93	0.963
c1_h16(q_c)	1.81	0.965
h12_o15(c_c)	1.76	0.941
c9_c13(q_c)	1.39	0.970
c9_c13(c_c)	1.28	0.973
c1_c13(q_c)	1.28	0.972
c11_c13(c_c)	1.28	0.981
c1_c13(c_c)	1.26	0.973
c7_h16(q_c)	1.05	0.980
h10_o14(c_c)	0.99	0.962
c3_h16(q_c)	0.99	0.981
.	.	.
c9_o14(q_c)	-0.75	-0.987
c1_o14(c_c)	-0.99	-0.973
c9_o14(c_c)	-1.02	-0.973
c11_o15(c_c)	-1.07	-0.980
c11_o14(c_c)	-1.09	-0.978
h2_c13(c_c)	-1.09	-0.966
c1_o15(c_c)	-1.14	-0.970
c9_o15(c_c)	-1.14	-0.970
h10_c13(c_c)	-1.20	-0.964
h12_h16(c_c)	-1.23	-0.942
c5_o15(q_c)	-1.24	-0.962
c3_o15(q_c)	-1.35	-0.965
c7_o15(q_c)	-1.38	-0.965
c11_o14(q_c)	-1.64	-0.960
c1_o15(q_c)	-1.66	-0.969
c9_o15(q_c)	-1.70	-0.969
c11_o15(q_c)	-1.84	-0.972
h12_c13(c_c)	-2.37	-0.937

TERM	REG
C_6_h16(q_c)	9.14
C_6_c13(q_c)	6.33
h12_o14(c_c)	2.21
h12_o15(c_c)	1.76
c11_c13(c_c)	1.28
c1_c13(c_c)	1.26
h10_o14(c_c)	0.99
.	.
c1_o14(c_c)	-0.99
c9_o14(c_c)	-1.02
c11_o15(c_c)	-1.07
c11_o14(c_c)	-1.09
h2_c13(c_c)	-1.09
c1_o15(c_c)	-1.14
c9_o15(c_c)	-1.14
h10_c13(c_c)	-1.20
h12_h16(c_c)	-1.23
h12_c13(c_c)	-2.37
C_6_o14(q_c)	-3.27
C_6_o15(q_c)	-9.17

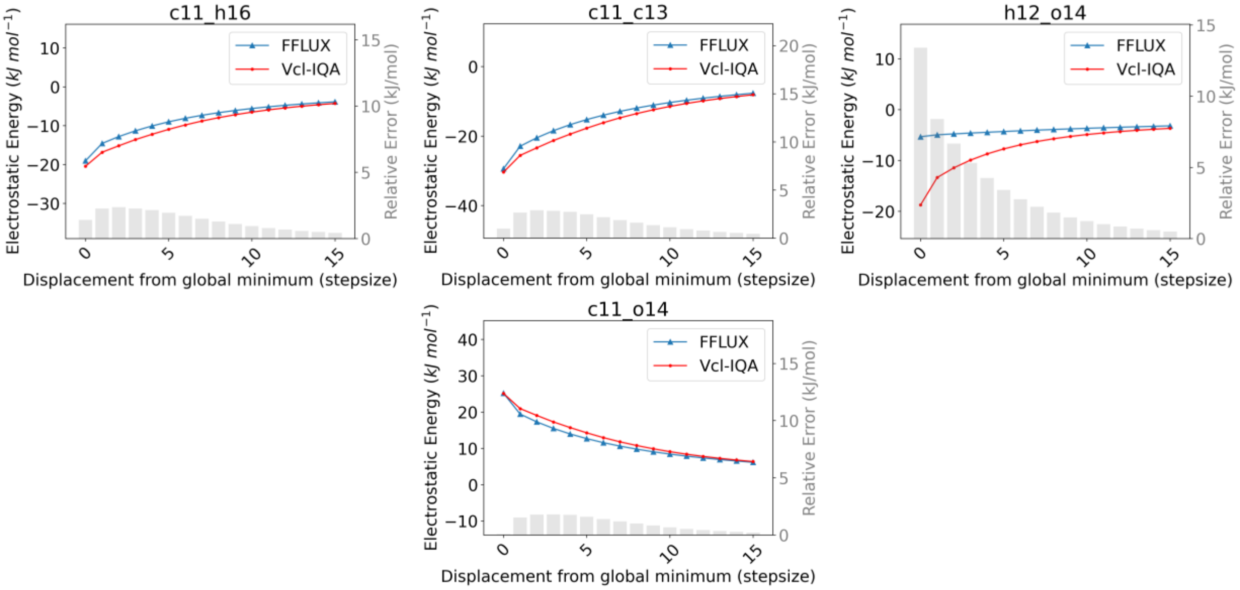

Figure S19. Convergence graphs of the monomeric energies used in REG-MULTI compared to the electrostatic IQA energies for the interactions between the closest atoms in the AcOH…benzene complex. V_{cl} refers to the exact electrostatic energy obtained by 6D integration, while "FFLUX" refers to this energy approximated by multipole expansion at L'=4 truncation, while the grey histogram represents the absolute energy difference. Note that energy electrostatic energy components are only summed up to L=5.

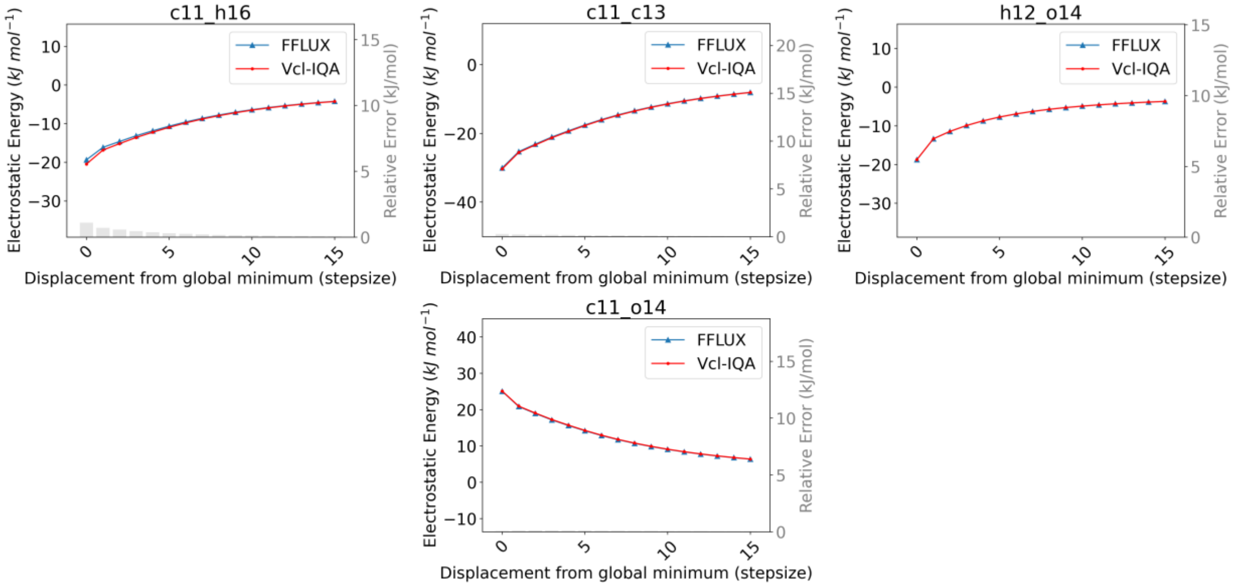

Figure S20. Convergence graphs of the dimeric energies used in REG-MULTI compared to the electrostatic IQA energies for the interactions between the closest atoms in the AcOH…benzene complex. V_{cl} refers to the exact electrostatic energy obtained by 6D integration, while "FFLUX" refers to this energy approximated by multipole expansion at L'=4 truncation, while the grey histogram represents the absolute energy difference. Note that energy electrostatic energy components are only summed up to L=5.

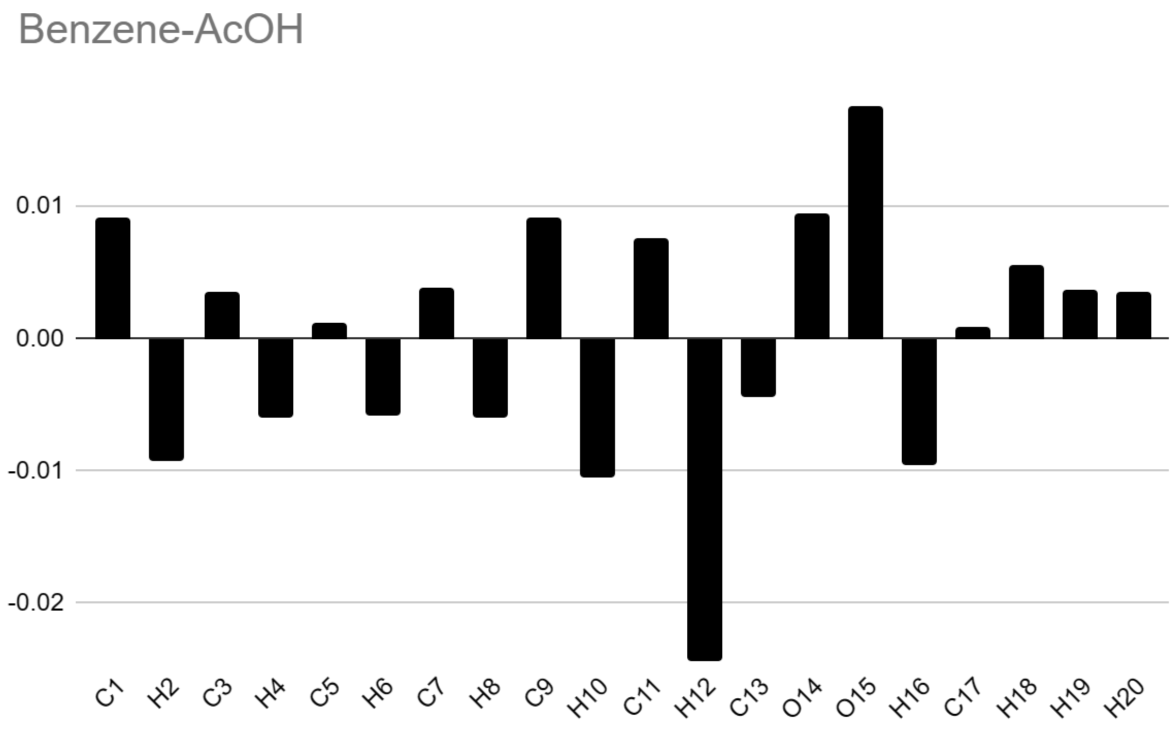

Figure S21. The difference (dimer minus monomer) between the atomic charges (in atomic units of electronic charge, e) of the REG-MULTI analysis (monomeric result) and the atomic charges of the energy minimum's geometry of the REG-MULTI analysis (dimeric result) in the AcOH…benzene complex.

S2.4 MeNH$_2$…benzene complex

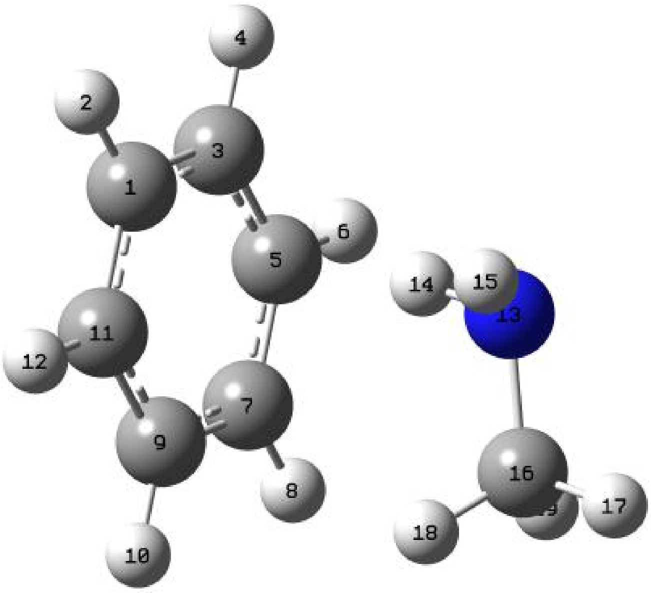

Figure S22. The geometry of the MeNH$_2$…benzene complex's energy minimum and its atom labelling.

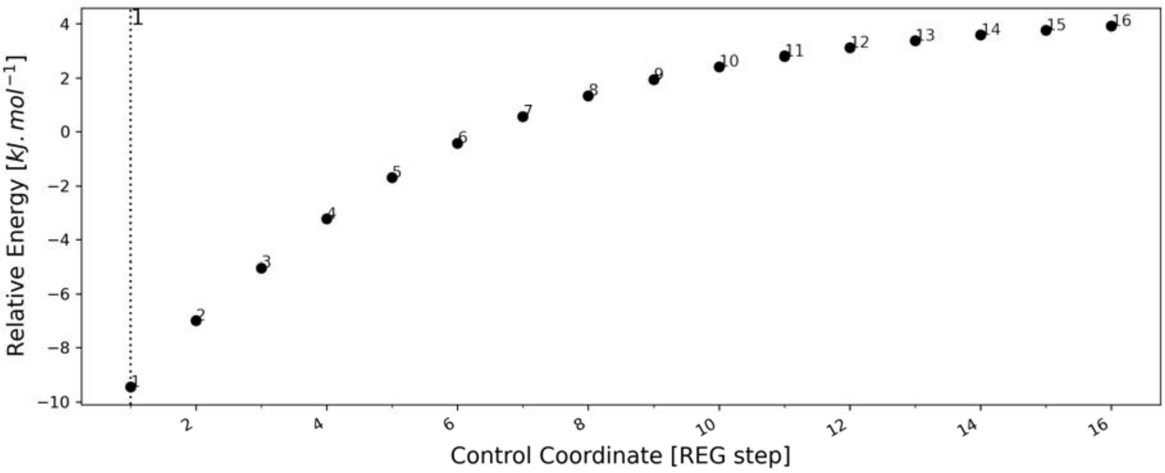

Figure S23. PES of the MeNH2…benzene complex. Total wavefunction energy curve considered for the REG-IQA analysis. The Y-axis corresponds to energy relative to the average value over all energies, while the X-axis shows the control coordinate steps starting from the complex minimum (dotted vertical line).

Table S7. Largest positive and negative REG values of the REG-IQF analysis of the $MeNH_2$…benzene complex.

**TERM**	**REG**
V_{cl}(H6,n13)	2.30
V_{xc}(C6,h14)	1.75
V_{cl}(C6,h14)	1.67
V_{cl}(n13,h14)	1.28
V_{cl}(C6,c16)	1.02
V_{xc}(C6,n13)	1.00
V_{disp}	1.00
...	...
E_{intra}(n13)	-0.44
V_{cl}(h14,c16)	-0.88
E_{intra}(h14)	-1.05
V_{cl}(H6,h14)	-1.18
V_{xc}(n13,h14)	-1.32
V_{cl}(C6,n13)	-3.08

Table S8. Largest positive and negative REG values for the $MeNH_2$…benzene complex: (left) the monomeric REG-MULTI analysis, (centre) the dimeric REG-MULTI analysis, and (right) the dimeric REG-MULTI analysis modified to show the sum of quadrupole(q)_charge(c) REG values.

TERM	REG	R
c3_h14(q_c)	2.12	0.992
c5_h14(q_c)	2.10	0.994
c1_h14(q_c)	1.59	0.997
c7_h14(q_c)	1.57	0.999
c11_h14(q_c)	1.24	1.000
c9_c16(q_c)	1.22	0.992
c9_h14(q_c)	1.20	1.000
c7_c16(q_c)	1.17	0.994
.	.	.
c11_n13(c_c)	-0.78	-0.996
c3_n13(c_c)	-0.79	-0.996
c1_n13(c_c)	-0.80	-0.996
c5_n13(c_c)	-0.81	-0.996
c7_n13(c_c)	-0.81	-0.996
c9_n13(c_c)	-0.82	-0.996
c1_n13(q_c)	-2.23	-0.998
c11_n13(q_c)	-2.24	-0.998
c9_n13(q_c)	-2.47	-0.997
c3_n13(q_c)	-2.54	-0.997
c7_n13(q_c)	-2.80	-0.997
c5_n13(q_c)	-2.85	-0.997

TERM	REG	R
c3_h14(q_c)	1.82	0.973
c5_h14(q_c)	1.80	0.978
c1_h14(q_c)	1.39	0.982
c7_h14(q_c)	1.35	0.990
h6_n13(c_c)	1.20	0.986
h4_n13(c_c)	1.08	0.987
c11_h14(q_c)	1.06	0.992
c9_h14(q_c)	1.03	0.995
c9_c16(q_c)	0.99	0.978
c7_c16(q_c)	0.95	0.981
.	.	.
c9_n13(c_c)	-0.64	-0.968
c7_n13(c_c)	-0.79	-0.993
c5_n13(c_c)	-0.81	-0.996
c11_n13(c_c)	-0.91	-0.996
c3_n13(c_c)	-1.01	-0.995
c1_n13(c_c)	-1.13	-0.988
c11_n13(q_c)	-1.87	-0.987
c1_n13(q_c)	-1.90	-0.987
c9_n13(q_c)	-2.02	-0.987
c3_n13(q_c)	-2.13	-0.986
c7_n13(q_c)	-2.31	-0.985
c5_n13(q_c)	-2.37	-0.985

TERM	REG
C_6_h14(q_c)	8.46
C_6_c16(q_c)	3.22
h6_n13(c_c)	1.20
h4_n13(c_c)	1.08
.	.
c9_n13(c_c)	-0.64
c7_n13(c_c)	-0.79 3
c5_n13(c_c)	-0.81
c11_n13(c_c)	-0.91
c3_n13(c_c)	-1.01
c1_n13(c_c)	-1.13
C_6_n13(q_c)	-12.60

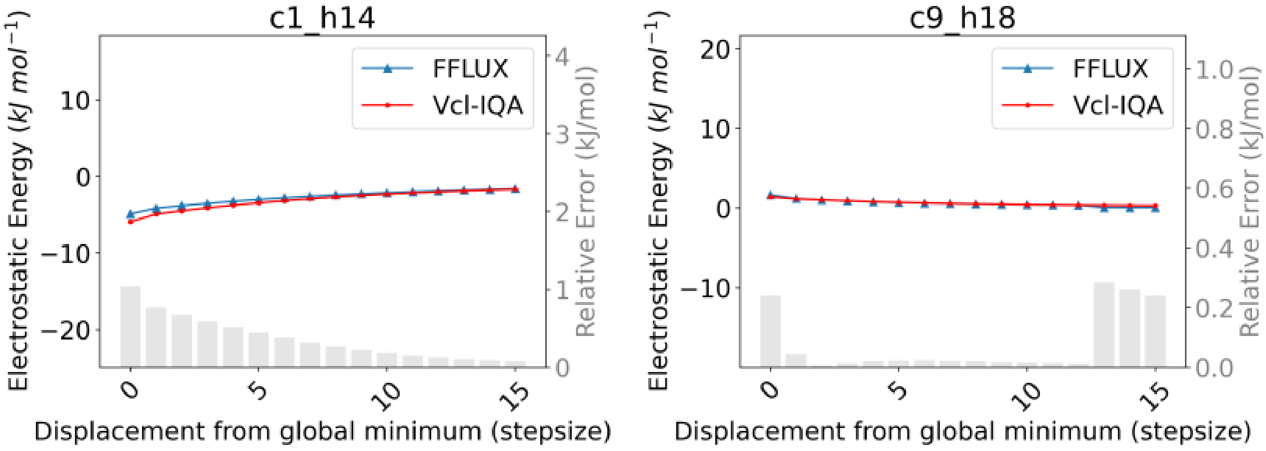

Figure S24. Convergence graphs of the monomeric energies used in REG-MULTI compared to the electrostatic IQA energies for the interactions between the closest atoms in the $MeNH_2$…benzene complex. V_{cl} refers to the exact electrostatic energy obtained by 6D integration, while "FFLUX" refers to this energy approximated by multipole expansion at L'=4 truncation, while the grey histogram represents the absolute energy difference. Note that energy electrostatic energy components are only summed up to L=5.

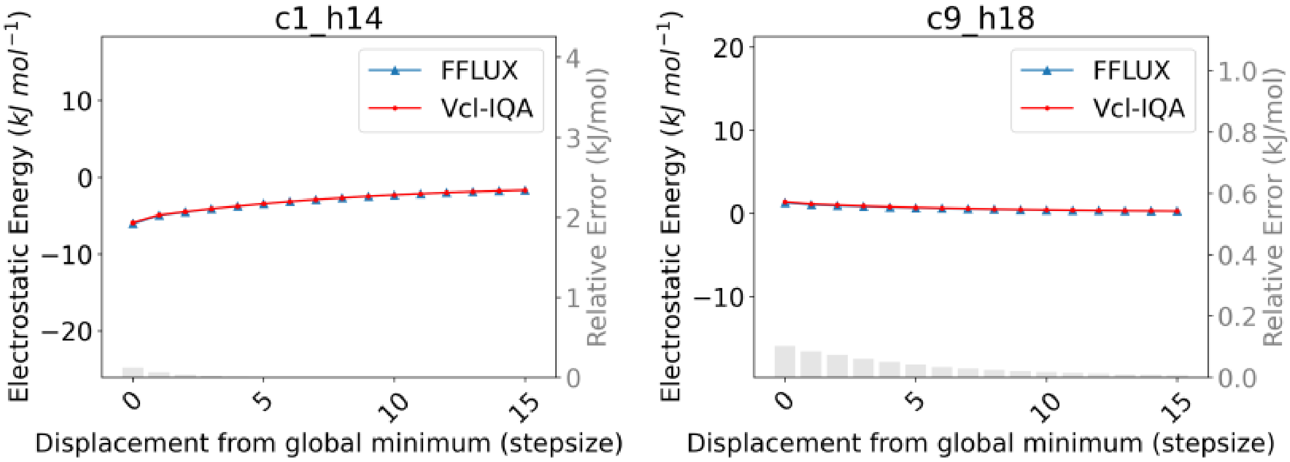

Figure S25. Convergence graphs of the dimeric energies used in REG-MULTI compared to the electrostatic IQA energies for the interactions between the closest atoms in the $MeNH_2$…benzene complex. V_{cl} refers to the exact electrostatic energy obtained by 6D integration, while "FFLUX" refers to this energy approximated by multipole expansion at L'=4 truncation, while the grey histogram represents the absolute energy difference. Note that energy electrostatic energy components are only summed up to L=5.

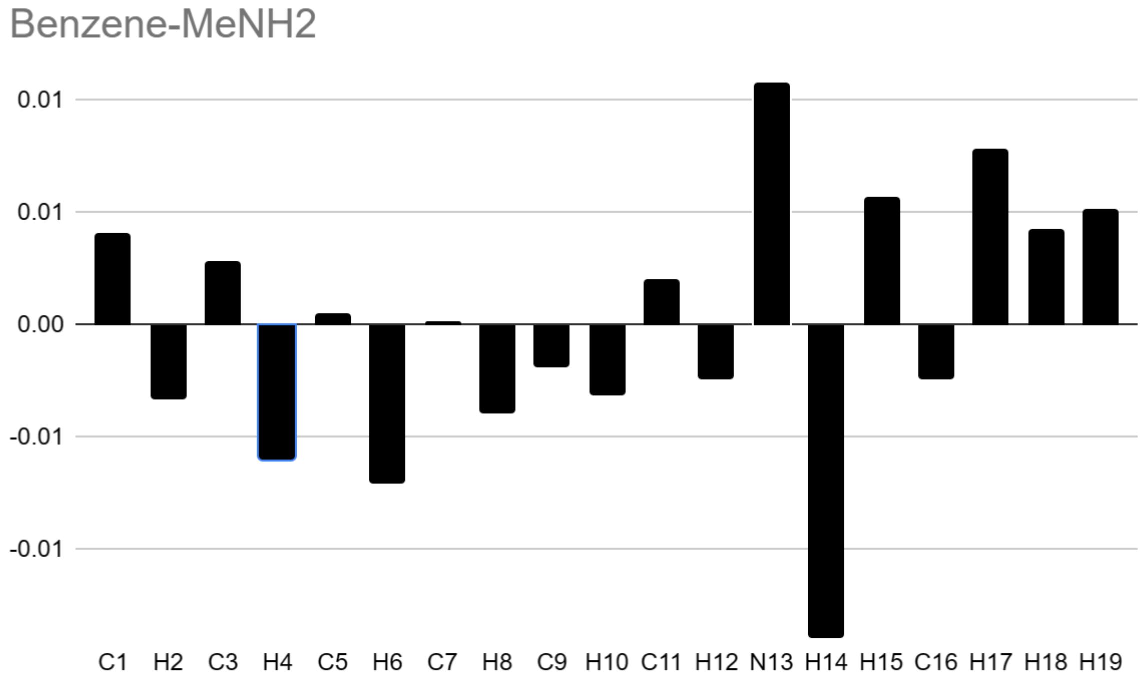

Figure S26. The difference (dimer minus monomer) between the atomic charges (in atomic units of electronic charge, e) of the REG-MULTI analysis (monomeric result) and the atomic charges of the energy minimum's geometry of the REG-MULTI analysis (dimeric result) in the $MeNH_2$…benzene complex.

S2.5 NMA…benzene complex

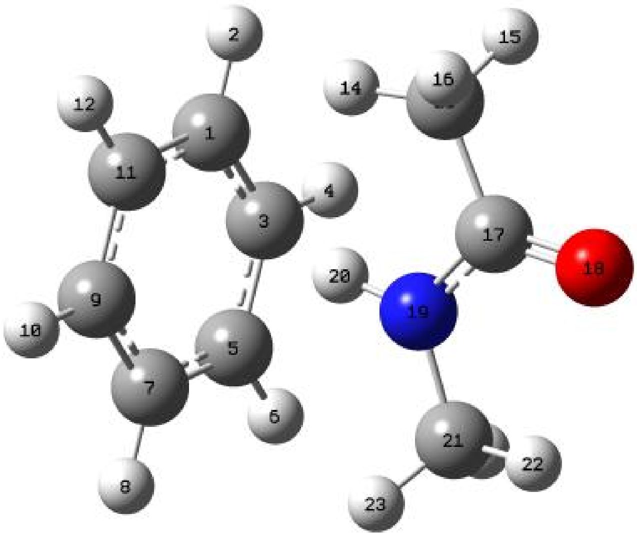

Figure S27. The geometry of the NMA…benzene complex's energy minimum and its atom labelling.

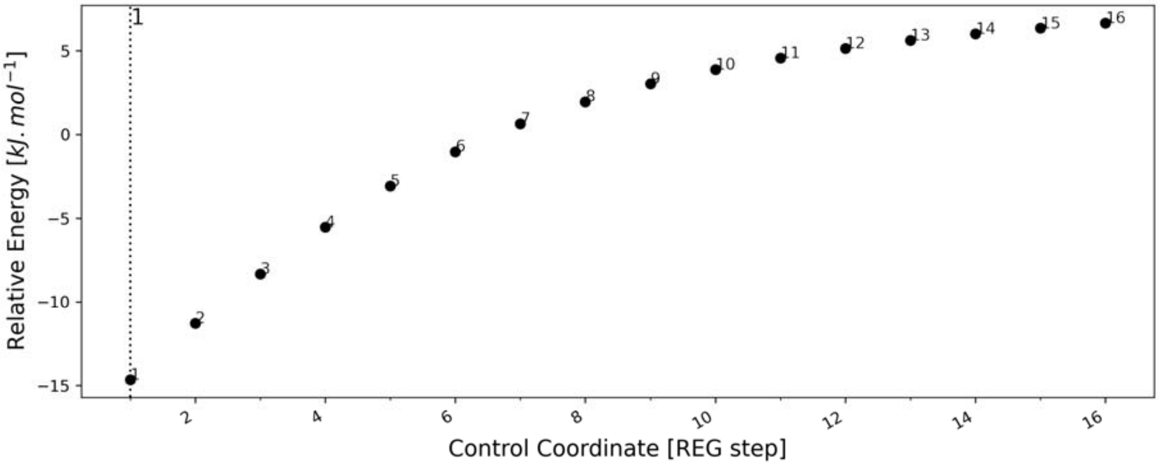

Figure S28. PES of the NMA…benzene complex. Total wavefunction energy curve considered for the REG-IQA analysis. The Y-axis corresponds to energy relative to the average value over all energies, while the X-axis shows the control coordinate steps starting from the complex minimum (dotted vertical line).

Table S9. Largest positive and negative REG values of the REG-IQF analysis of the NMA…benzene complex.

**TERM**	**REG**
$V_{cl}(H_6,n19)$	2.22
$V_{cl}(C_6,c17)$	2.21
$V_{cl}(C_6,h20)$	1.56
$V_{xc}(C_6,h20)$	1.15
$V_{cl}(n19,h20)$	1.11
V_{disp}	0.87
…	…
$E_{intra}(h20)$	-0.76
$V_{xc}(n19,h20)$	-0.96
$V_{cl}(H_6,h20)$	-1.10
$V_{cl}(C_6,o18)$	-1.25
$V_{cl}(H_6,c17)$	-1.88
$V_{cl}(C_6,n19)$	-2.90

Table S10. Largest positive and negative REG values for the NMA…benzene complex: (left) the monomeric REG-MULTI analysis, (centre) the dimeric REG-MULTI analysis, and (right) the dimeric REG-MULTI analysis modified to show the sum of quadrupole(q)_charge(c) REG values.

TERM	REG	R
c1_c17(q_c)	1.62	0.938
c11_h20(q_c)	1.53	0.988
c11_c17(q_c)	1.47	0.928
c9_h20(q_c)	1.36	0.990
h8_n19(c_c)	1.32	0.801
h6_n19(c_c)	1.31	0.801
h4_n19(c_c)	1.30	0.798
c1_h20(q_c)	1.30	0.990
h10_n19(c_c)	1.29	0.798
h12_n19(c_c)	1.29	0.795
c3_c17(q_c)	1.29	0.911
h2_n19(c_c)	1.27	0.795
c9_c17(q_c)	1.07	0.882
c7_h20(q_c)	1.02	0.991
c3_h20(q_c)	1.02	0.989
c5_c17(q_c)	0.94	0.855
c5_h20(q_c)	0.88	0.988
h12_n19(d_c)	0.86	0.938
c7_c17(q_c)	0.86	0.837
.	.	.
c3_n19(c_c)	-1.44	-0.817
c5_n19(c_c)	-1.44	-0.820
c11_n19(c_c)	-1.44	-0.815
c7_n19(c_c)	-1.46	-0.819
c1_n19(c_c)	-1.48	-0.815
c9_n19(c_c)	-1.50	-0.817
c1_n19(q_c)	-2.23	-0.941
c11_n19(q_c)	-2.28	-0.941
c3_n19(q_c)	-2.39	-0.949
c9_n19(q_c)	-2.43	-0.948
c5_n19(q_c)	-2.53	-0.955
c7_n19(q_c)	-2.55	-0.955

TERM	REG	R
c1_c17(q_c)	1.18	0.965
c11_h20(q_c)	1.15	0.958
c11_c17(c_c)	1.14	0.969
c11_c17(q_c)	1.06	0.972
c9_h20(q_c)	1.02	0.963
c1_h20(q_c)	0.97	0.964
c3_c17(q_c)	0.91	0.970
h4_n19(c_c)	0.88	0.966
c1_c17(c_c)	0.86	0.983
h10_n19(c_c)	0.86	0.962
h6_n19(c_c)	0.81	0.970
h8_n19(c_c)	0.79	0.970
h2_n19(c_c)	0.79	0.965
c7_h20(q_c)	0.77	0.971
c3_h20(q_c)	0.75	0.971
h12_n19(c_c)	0.74	0.964
c9_c17(q_c)	0.74	0.982
c9_c17(c_c)	0.73	0.990
c5_h20(q_c)	0.66	0.974
c5_c17(q_c)	0.63	0.982
h4_o18(c_c)	0.60	0.968
c11_h20(c_c)	0.57	0.957
c7_c17(q_c)	0.57	0.986
.	.	.
c9_n19(c_c)	-0.82	-0.986
c1_n19(c_c)	-0.86	-0.981
h2_c17(c_c)	-0.89	-0.963
h4_c17(c_c)	-0.90	-0.968
c11_n19(c_c)	-1.15	-0.967
c1_n19(q_c)	-1.26	-0.974
c11_n19(q_c)	-1.27	-0.976
c3_n19(q_c)	-1.36	-0.964
c9_n19(q_c)	-1.39	-0.971
c5_n19(q_c)	-1.45	-0.959
c7_n19(q_c)	-1.47	-0.962

TERM	REG
C_6_h20(q_c)	5.32
C_6_c17(q_c)	5.08
c11_c17(c_c)	1.14
h4_n19(c_c)	0.88
c1_c17(c_c)	0.86
h10_n19(c_c)	0.86
h6_n19(c_c)	0.81
h8_n19(c_c)	0.79
h2_n19(c_c)	0.79
h12_n19(c_c)	0.74
c9_c17(c_c)	0.73
h4_o18(c_c)	0.60
c11_h20(c_c)	0.57
.	.
c9_n19(c_c)	-0.82
c1_n19(c_c)	-0.86
h2_c17(c_c)	-0.89
h4_c17(c_c)	-0.90
c11_n19(c_c)	-1.15
C_6_o18(q_c)	-2.23
C_6_n19(q_c)	-8.21

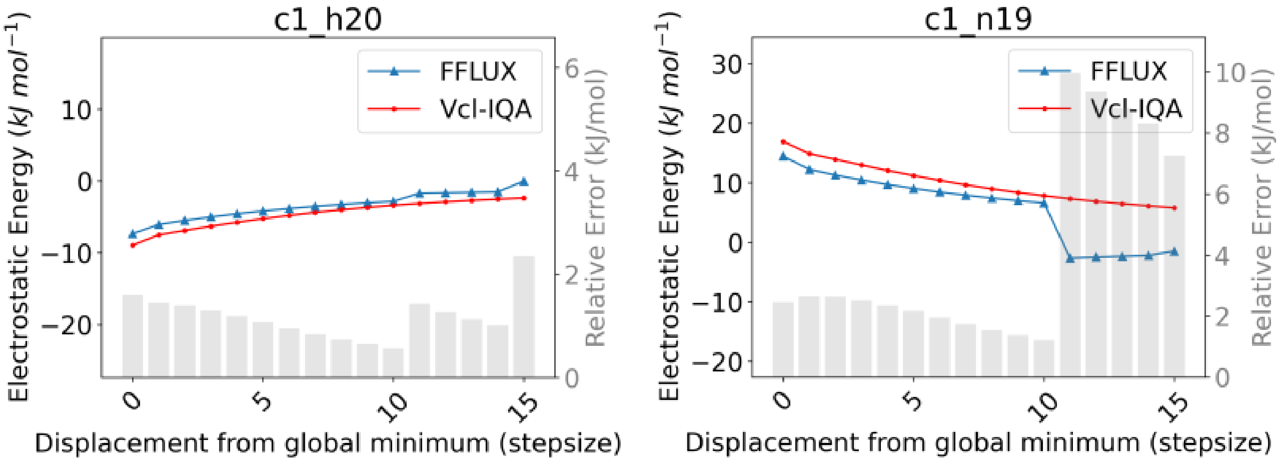

Figure S29. Convergence graphs of the monomeric energies used in REG-MULTI compared to the electrostatic IQA energies for the interactions between the closest atoms in the benzene…NMA complex. V_{cl} refers to the exact electrostatic energy obtained by 6D integration, while "FFLUX" refers to this energy approximated by multipole expansion at L'=4 truncation, while the grey histogram represents the absolute energy difference. Note that energy electrostatic energy components are only summed up to L=5.

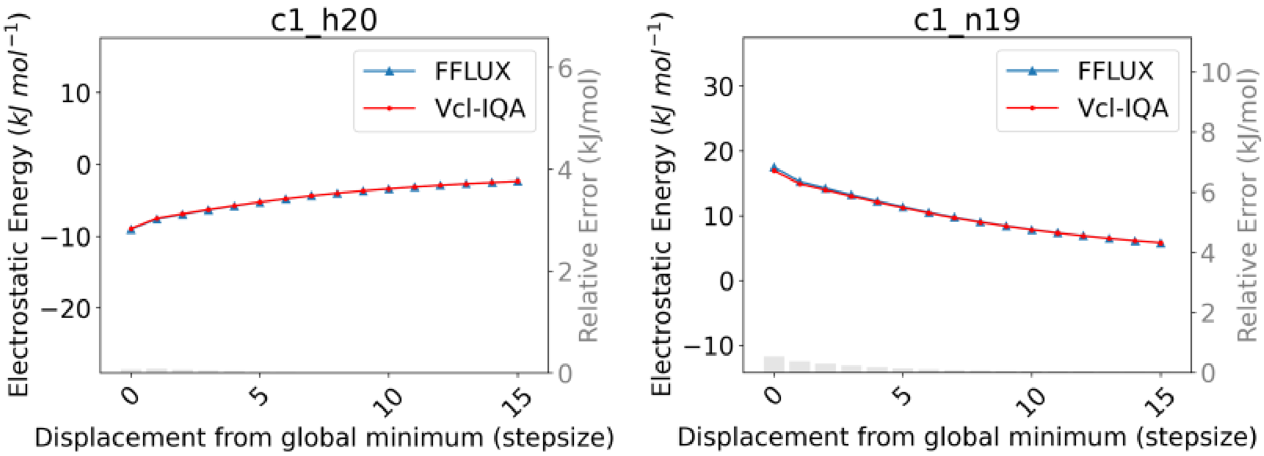

Figure S30. Convergence graphs of the dimeric energies used in REG-MULTI compared to the electrostatic IQA energies for the interactions between the closest atoms in the benzene…NMA complex. V_{cl} refers to the exact electrostatic energy obtained by 6D integration, while "FFLUX" refers to this energy approximated by multipole expansion at L'=4 truncation, while the grey histogram represents the absolute energy difference. Note that energy electrostatic energy components are only summed up to L=5.

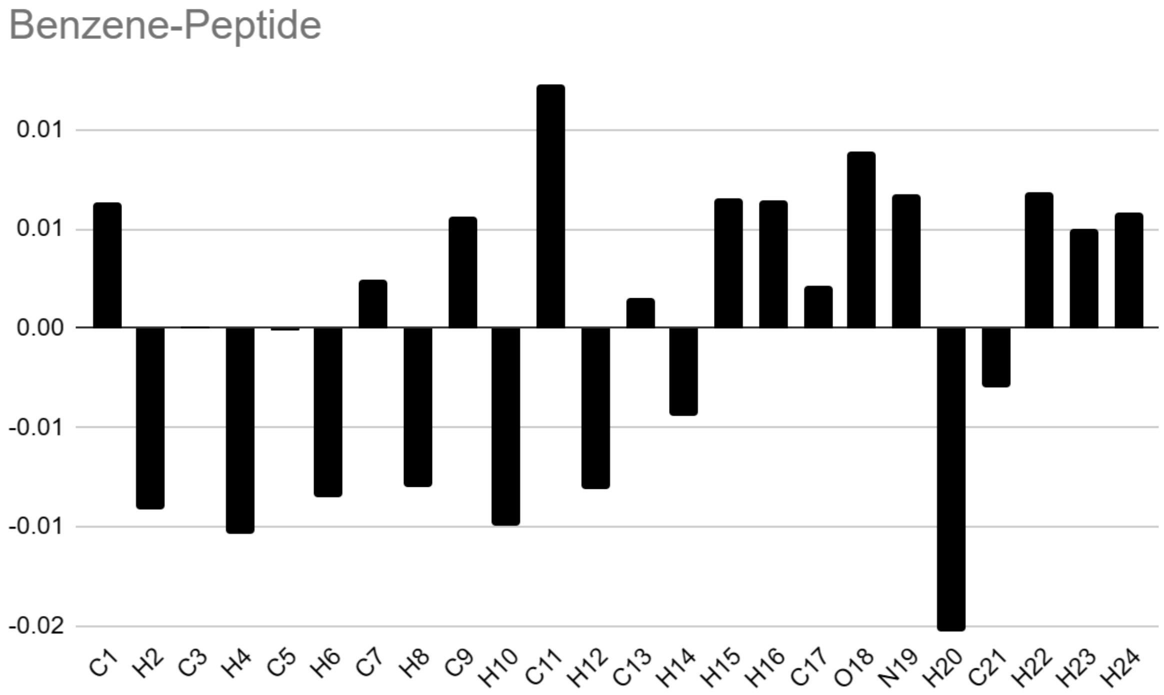

Figure S31. The difference (dimer minus monomer) between the atomic charges (in atomic units of electronic charge, e) of the REG-MULTI analysis (monomeric result) and the atomic charges of the energy minimum's geometry of the REG-MULTI analysis (dimeric result) in the NMA…benzene complex.

S2.6 Water…benzene complex

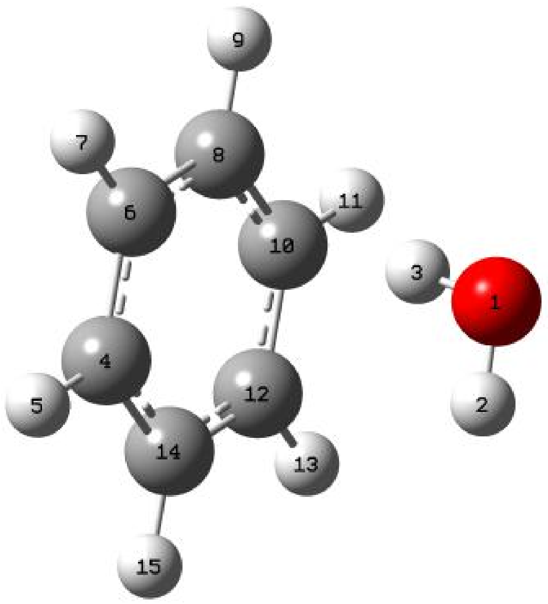

Figure S32. The geometry of the water…benzene complex's energy minimum and its atom labelling.

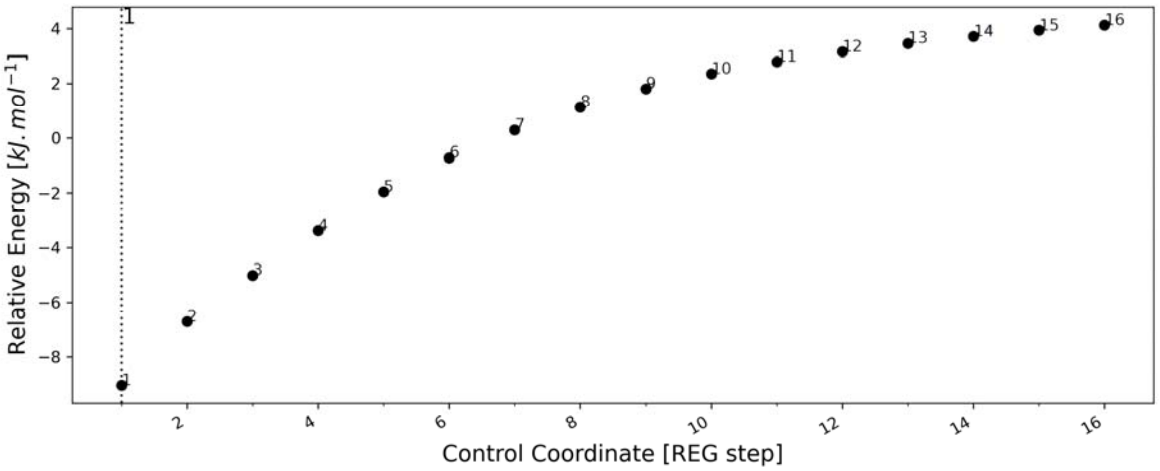

Figure S33. PES of the water…benzene complex. Total wavefunction energy curve considered for the REG-IQA analysis. The Y axis corresponds to energy relative to the average value over all energies, while the X axis shows the control coordinate steps starting from the complex minimum (dotted vertical line).

Table S11. Largest positive and negative REG values of the REG-IQF analysis of the water…benzene complex.

TERM	REG
V_{cl}(h3,C_6)	3.58
V_{cl}(o1,H_6)	3.53
V_{cl}(h2,C_6)	2.28
V_{cl}(o1,h3)	2.19
V_{xc}(o1,C_6)	1.50
V_{xc}(h3,C_6)	1.49
…	…
V_{cl}(h2,H_6)	-1.60
E_{intra}(o1)	-1.65
V_{xc}(o1,h3)	-1.68
V_{cl}(h3,H_6)	-2.41
V_{cl}(o1,C_6)	-4.94

Table S12. Largest positive and negative REG values for the water…benzene complex: (left) the monomeric REG-MULTI analysis, (centre) the dimeric REG-MULTI analysis, and (right) the dimeric REG-MULTI analysis modified to show the sum of quadrupole(q)_charge(c) REG values.

TERM	REG	R
h3_c8(c_q)	2.60	0.989
h3_c10(c_q)	1.83	0.996
h3_c6(c_q)	1.83	0.996
h2_c14(c_q)	1.06	0.993
h3_c12(c_q)	0.96	1.000
h3_c4(c_q)	0.94	1.000
h2_c12(c_q)	0.87	0.997
h2_c4(c_q)	0.86	0.997
h3_c14(c_q)	0.71	0.997
.	.	.
o1_c14(c_q)	-1.19	-0.999
o1_c4(c_q)	-1.34	-0.999
o1_c12(c_q)	-1.35	-0.999
o1_c6(c_q)	-1.76	-0.998
o1_c10(c_q)	-1.76	-0.998
o1_c8(c_q)	-2.02	-0.997

TERM	REG	R
h3_c8(c_q)	2.34	0.976
h3_c10(c_q)	1.68	0.998
h3_c6(c_q)	1.57	0.956
o1_h9(c_c)	0.94	0.983
h2_c14(c_q)	0.93	0.971
o1_h11(c_c)	0.93	0.988
o1_h7(c_c)	0.91	0.981
h3_c8(c_c)	0.90	0.983
h3_c12(c_q)	0.87	0.999
h3_c4(c_q)	0.84	0.971
h2_c12(c_q)	0.77	0.995
h2_c4(c_q)	0.76	0.970
o1_h13(c_c)	0.71	0.990
o1_h5(c_c)	0.70	0.985
h3_c14(c_q)	0.63	0.994
.	.	.
o1_c14(c_q)	-1.08	-0.983
o1_c12(c_q)	-1.21	-0.994
o1_c4(c_q)	-1.22	-0.971
o1_c8(c_c)	-1.25	-0.986
o1_c10(c_q)	-1.56	-0.996
o1_c6(c_q)	-1.57	-0.974
o1_c8(c_q)	-1.81	-0.988

TERM	REG
h3_C_6(c_q)	7.93
h2_C_6(c_q)	3.91
o1_h9(c_c)	0.94
o1_h11(c_c)	0.93
o1_h7(c_c)	0.91
o1_h13(c_c)	0.71
o1_h5(c_c)	0.70
.	.
o1_c8(c_c)	-1.25
o1_C_6(c_q)	-8.45

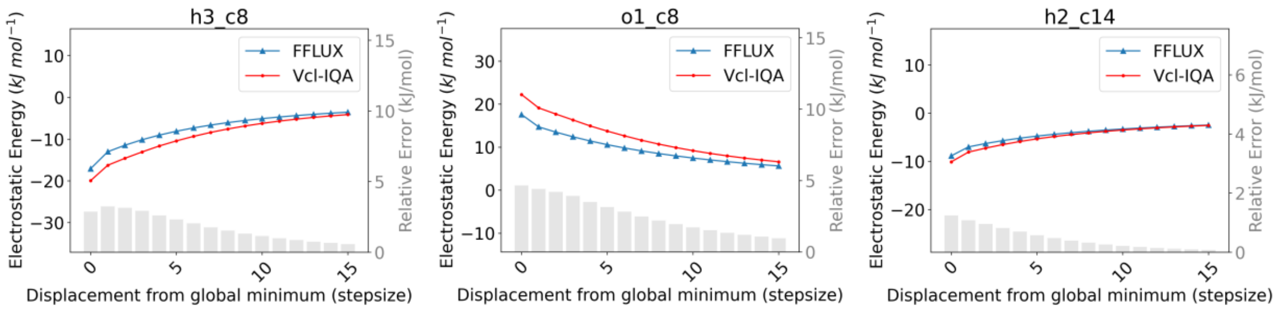

Figure S34. Convergence graphs of the monomeric energies used in REG-MULTI compared to the electrostatic IQA energies for the interactions between the closest atoms in the water…benzene complex. V_{cl} refers to the exact electrostatic energy obtained by 6D integration, while "FFLUX" refers to this energy approximated by multipole expansion at L'=4 truncation, while the grey histogram represents the absolute energy difference. Note that energy electrostatic energy components are only summed up to L=5.

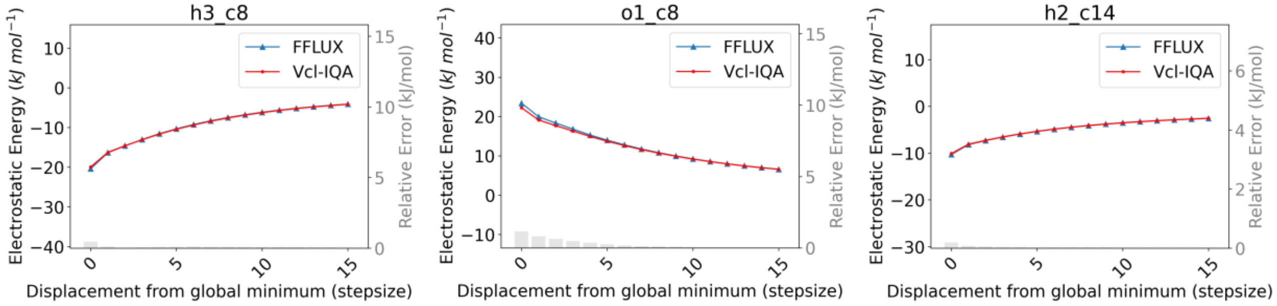

Figure S35. Convergence graphs of the dimeric energies used in REG-MULTI compared to the electrostatic IQA energies for the interactions between the closest atoms in the water…benzene complex. V_{cl} refers to the exact electrostatic energy obtained by 6D integration, while "FFLUX" refers to this energy approximated by multipole expansion at L'=4 truncation, while the grey histogram represents the absolute energy difference. Note that energy electrostatic energy components are only summed up to L=5.

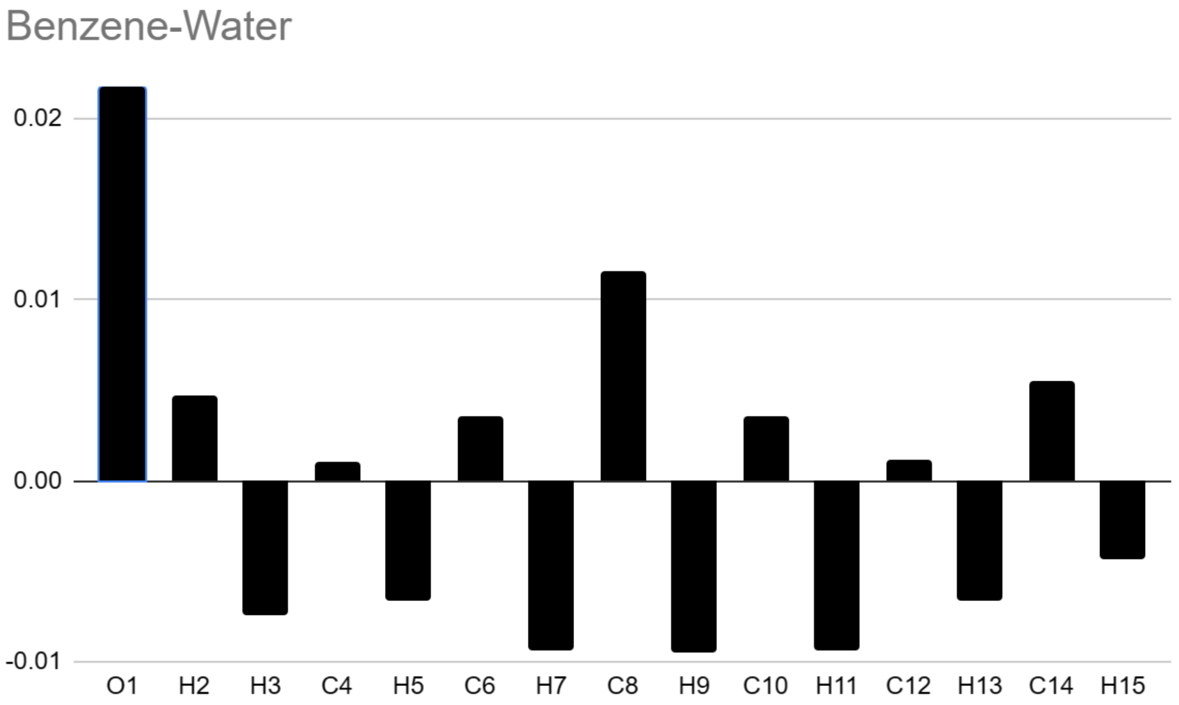

Figure S36. The difference (dimer minus monomer) between the atomic charges (in atomic units of electronic charge, e) of the REG-MULTI analysis (monomeric result) and the atomic charges of the energy minimum's geometry of the REG-MULTI analysis (dimeric result) in the water…benzene complex.

Note that only REG-IQA analyses are presented for the two CH…π complexes ethane…benzene and ethane…benzene because electrostatics play a negligible role. However, for the ethyne…benzene complex, both REG-IQA and REG-FFLUX analysis will be shown because interesting electrostatic effects occur due to the acidic nature of the ethyne monomer.

S2.7 Ethane…benzene complex

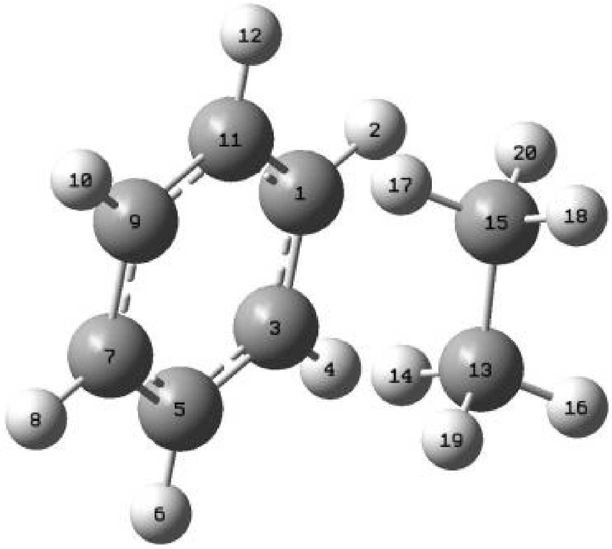

Figure S37. The geometry of the ethane…benzene complex's energy minimum and its atom labelling.

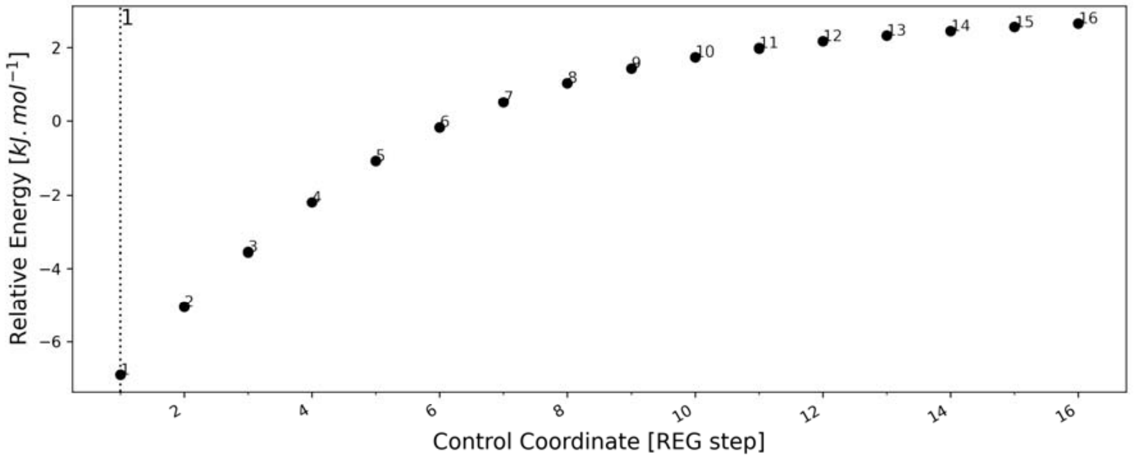

Figure S38. PES of the ethane…benzene complex. Total wavefunction energy curve considered for the REG-IQA analysis. The Y axis corresponds to energy relative to the average value over all energies, while the X axis shows the control coordinate steps starting from the complex minimum (dotted vertical line).

Table S13. Largest positive and negative REG values of the REG-IQF analysis and the corresponding energies at the energy minimum's geometry and at "infinite" separation for the ethane…benzene complex. The energies are in kJ/mol.

TERM	REG	Energy at energy minimum	Energy at largest monomer separation	delta
$V_{xc}(C_6,h14)$	2.53	-29.95	-0.10	29.86
V_{disp}	1.32	-78.61	-64.41	14.21
$V_{xc}(C_6,c13)$	0.76	-9.23	-0.02	9.21
$V_{xc}(C_6,c15)$	0.53	-6.51	-0.01	6.49
$V_{xc}(C_6,h17)$	0.48	-5.70	-0.02	5.68
$V_{xc}(C_6,h20)$	0.45	-5.36	-0.02	5.34
...	...	...	...	...
$E_{intra}(c11)$	-0.51	-98781.64	-98788.12	-6.48
$V_{xc}(c13,h14)$	-0.70	-732.21	-740.15	-7.94
$E_{intra}(h14)$	-1.47	-1190.51	-1207.45	-16.93

S2.8 Ethene…benzene complex

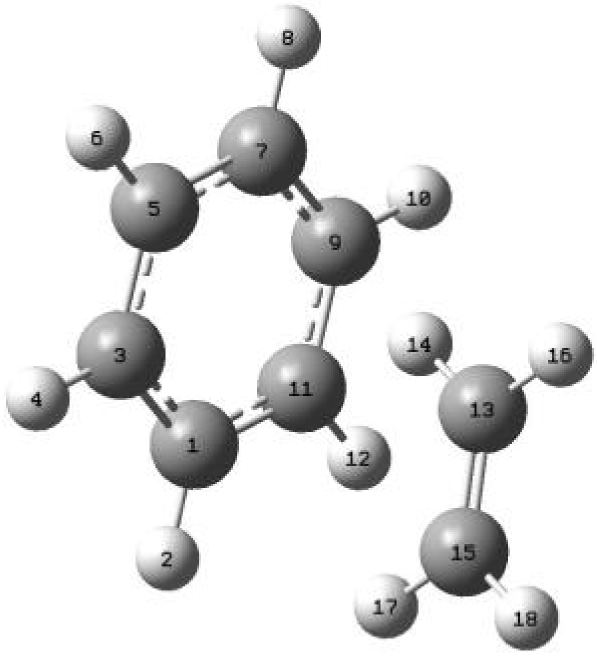

Figure S39. The geometry of the ethene…benzene complex's energy minimum and its atom labelling.

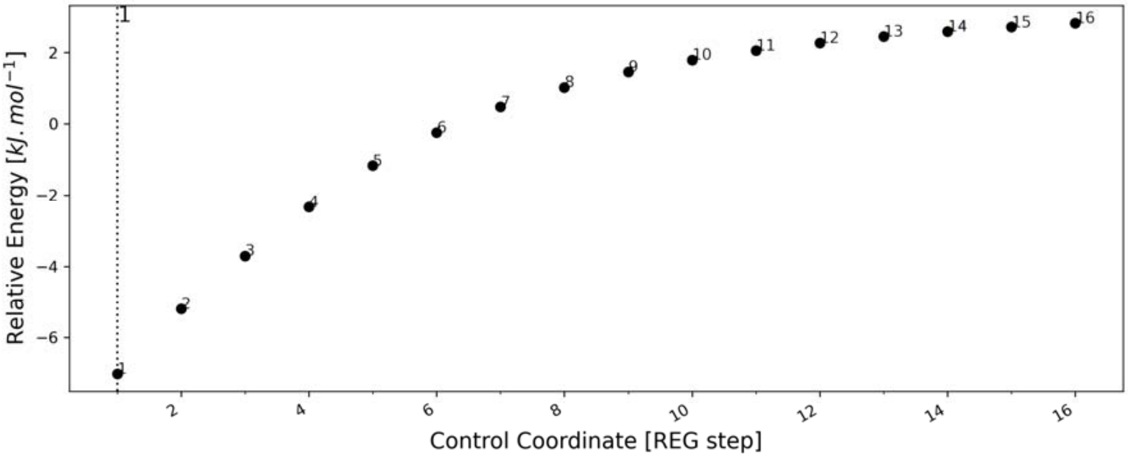

Figure S40. PES of the ethene…benzene complex. Total wavefunction energy curve considered for the REG-IQA analysis. The Y-axis corresponds to energy relative to the average value over all energies, while the X-axis shows the control coordinate steps starting from the complex minimum (dotted vertical line).

Table S14. Largest positive and negative REG values of the REG-IQF analysis and the corresponding energies at the energy minimum's geometry and at "infinite" separation for the ethene…benzene complex, the energies are in kJ/mol.

TERM	REG	Energy at energy minimum	Energy at largest monomer separation	delta
$V_{xc}(C_6,h14)$	2.49	-30.71	-0.09	30.62
V_{disp}	1.19	-73.66	-60.51	13.15
$V_{xc}(C_6,c13)$	0.92	-11.68	-0.03	11.66
$V_{xc}(C_6,h17)$	0.64	-7.82	-0.03	7.79
$V_{xc}(C_6,c15)$	0.42	-5.33	-0.02	5.31
...	...	...	...	...
$V_{xc}(c13,h14)$	-0.86	-741.66	-751.89	-10.23
$E_{intra}(h14)$	-1.48	-1185.76	-1203.25	-17.49

S2.9 Ethyne…benzene complex

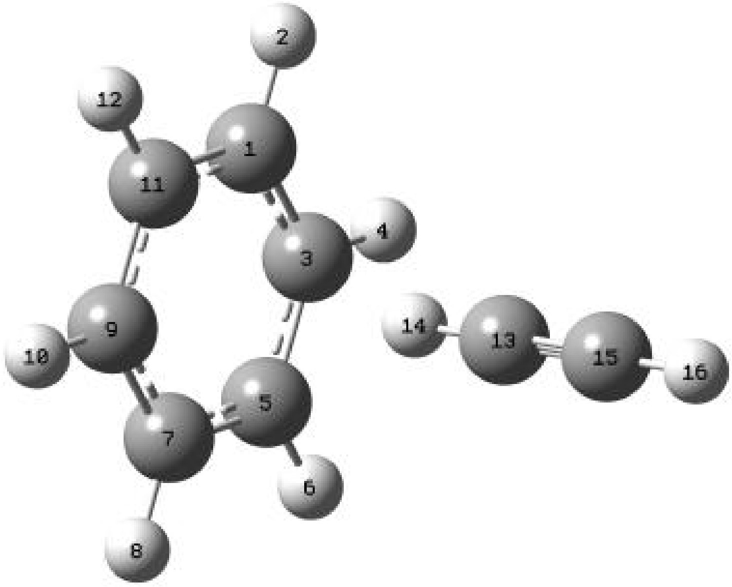

Figure S41. The geometry of the ethyne…benzene complex's energy minimum and its atom labelling.

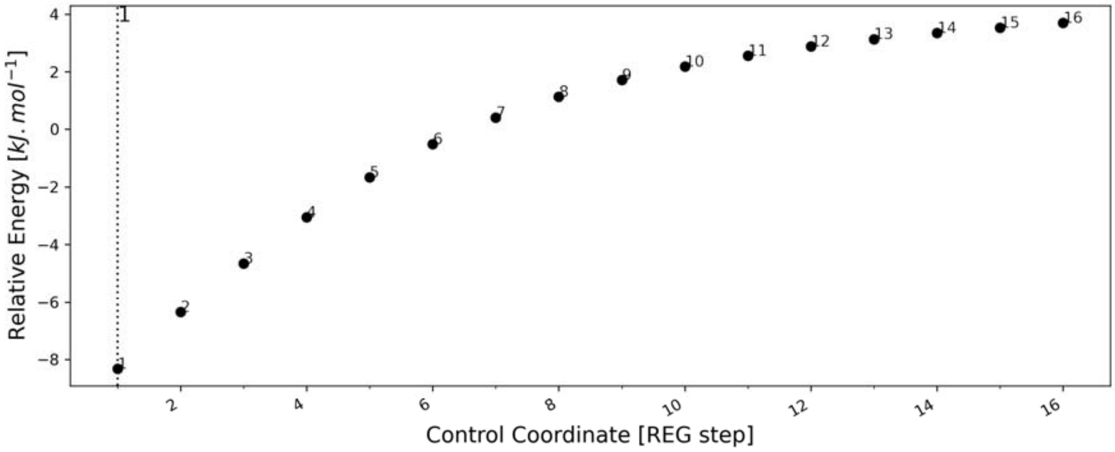

Figure S42. PES of the ethyne…benzene complex. Total wavefunction energy curve considered for the REG-IQA analysis. The Y-axis corresponds to energy relative to the average value over all energies, while the X-axis shows the control coordinate steps starting from the complex minimum (dotted vertical line).

Table S15. Largest positive and negative REG values of the REG-IQF analysis and the corresponding energies at the energy minimum's geometry and at "infinite" separation for the ethyne…benzene complex. The energies are in kJ/mol.

TERM	REG	Energy at energy minimum	Energy at largest monomer separation	delta
$V_{xc}(C_6,h14)$	2.31	-35.80	-0.09	35.70
$V_{cl}(c13,c15)$	1.38	295.37	317.47	22.10
$V_{cl}(C_6,h14)$	1.10	-19.53	-5.20	14.33
$V_{cl}(h14,c15)$	1.05	-14.83	0.12	14.96
$V_{xc}(C_6,c13)$	0.90	-16.15	-0.03	16.12
$V_{cl}(c15,h16)$	0.87	100.54	112.79	12.24
V_{disp}	0.79	-66.66	-56.00	10.66
$V_{cl}(H_6,c15)$	0.43	-9.40	-2.57	6.84
...	...	...	...	...
$V_{cl}(C_6,c15)$	-0.58	10.62	2.98	-7.64
$V_{cl}(H_6,h14)$	-0.77	14.09	3.84	-10.25
$V_{cl}(c13,h16)$	-0.93	16.84	3.91	-12.92
$E_{intra}(c13)$	-0.96	-98908.68	-98923.79	-15.11
$V_{xc}(c13,c15)$	-1.16	-1977.33	-1992.87	-15.53
$V_{xc}(c13,h14)$	-1.36	-727.92	-747.62	-19.70
$E_{intra}(h14)$	-1.40	-1136.62	-1157.26	-20.63

Table S16. Largest positive and negative REG values for the ethyne…benzene complex: (left) the monomeric REG-MULTI analysis, (centre) the dimeric REG-MULTI analysis, and (right) the dimeric REG-MULTI analysis modified to show the sum of quadrupole(q)_charge(c) REG values.

TERM	REG	R
c11_h14(q_c)	0.63	0.969
c9_h14(q_c)	0.62	0.969
c7_h14(q_c)	0.62	0.969
c3_h14(q_c)	0.62	0.969
c1_h14(q_c)	0.62	0.969
c5_h14(q_c)	0.61	0.969
c11_c13(q_q)	0.29	0.962
c9_c13(q_q)	0.29	0.963
c1_c13(q_q)	0.28	0.963
c3_c13(q_q)	0.28	0.963
c7_c13(q_q)	0.28	0.963
c5_c13(q_q)	0.28	0.964
.	.	.
h8_h14(d_c)	-0.21	-0.970
h4_h14(d_c)	-0.21	-0.970
h6_h14(d_c)	-0.21	-0.970
h2_h14(d_c)	-0.21	-0.970
h10_h14(d_c)	-0.21	-0.970
h12_h14(d_c)	-0.21	-0.970
c1_c13(q_c)	-0.30	-0.969
c3_c13(q_c)	-0.30	-0.969
c5_c13(q_c)	-0.30	-0.968
c9_c13(q_c)	-0.30	-0.969
c7_c13(q_c)	-0.30	-0.968
c11_c13(q_c)	-0.30	-0.969

TERM	REG	R
c3_h14(q_c)	0.60	0.894
c5_h14(q_c)	0.59	0.893
c1_h14(q_c)	0.59	0.894
c9_h14(q_c)	0.59	0.894
c11_h14(q_c)	0.59	0.895
c7_h14(q_c)	0.59	0.894
c11_c13(q_q)	0.25	0.892
c1_c13(q_q)	0.25	0.891
c9_c13(q_q)	0.25	0.891
c3_c13(q_q)	0.25	0.892
c5_c13(q_q)	0.24	0.892
c7_c13(q_q)	0.24	0.893
...		
h8_h14(d_c)	-0.20	-0.872
h8_h14(c_c)	-0.20	-0.886
h2_h14(d_c)	-0.20	-0.873
h2_h14(c_c)	-0.20	-0.887
h4_h14(d_c)	-0.20	-0.875
h10_h14(c_c)	-0.20	-0.884
h10_h14(d_c)	-0.20	-0.871
h4_h14(c_c)	-0.20	-0.888
h12_h14(c_c)	-0.20	-0.879
h12_h14(d_c)	-0.20	-0.863
h6_h14(c_c)	-0.21	-0.885
h6_h14(d_c)	-0.21	-0.870
c11_c15(q_c)	-0.21	-0.861
c1_c15(q_c)	-0.21	-0.860
c9_c15(q_c)	-0.21	-0.860
c7_c15(q_c)	-0.21	-0.860
c3_c15(q_c)	-0.22	-0.860
c5_c15(q_c)	-0.22	-0.859

TERM	REG
C_6_h14(q_c)	3.56
C_6_c13(q_q)	1.47
...	
h8_h14(d_c)	-0.20
h8_h14(c_c)	-0.20
h2_h14(d_c)	-0.20
h2_h14(c_c)	-0.20
h4_h14(d_c)	-0.20
h10_h14(c_c)	-0.20
h10_h14(d_c)	-0.20
h4_h14(c_c)	-0.20
h12_h14(c_c)	-0.20
h12_h14(d_c)	-0.20
h6_h14(c_c)	-0.21
h6_h14(d_c)	-0.21
C_6_c15(q_c)	-1.28

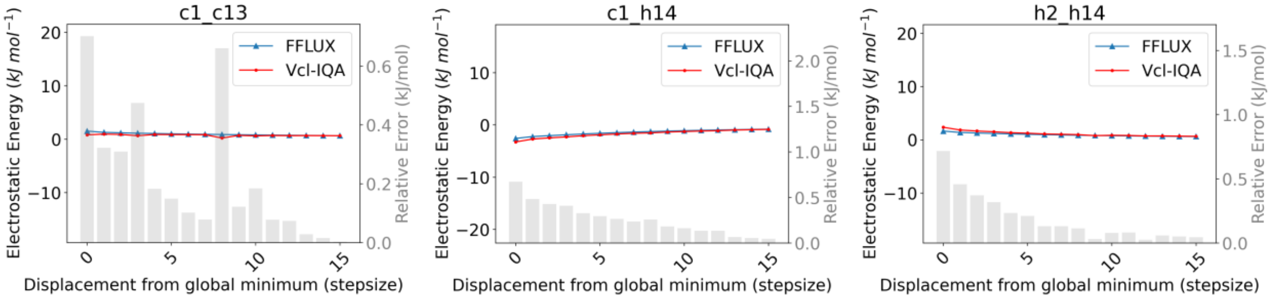

Figure S43. Convergence graphs of the monomeric energies used in REG-MULTI compared to the electrostatic IQA energies for the interactions between the closest atoms in the ethyne…benzene complex. V_{cl} refers to the exact electrostatic energy obtained by 6D integration, while "FFLUX" refers to this energy approximated by multipole expansion at L'=4 truncation, while the grey histogram represents the absolute energy difference. Note that energy electrostatic energy components are only summed up to L=5.

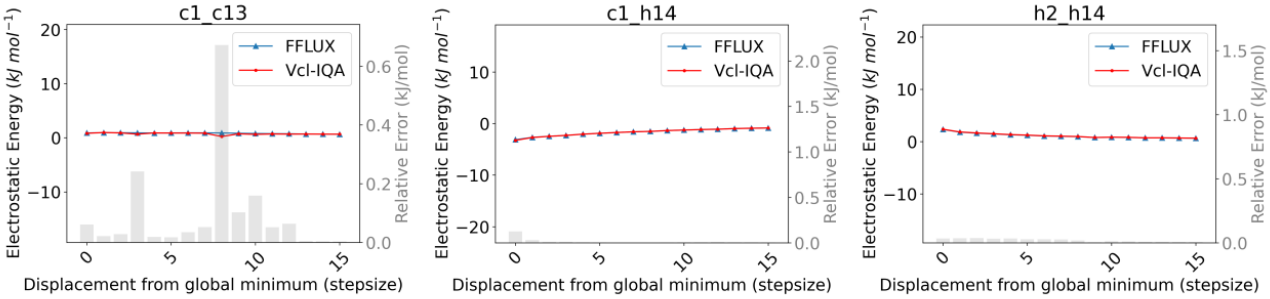

Figure S44. Convergence graphs of the dimeric energies used in REG-MULTI compared to the electrostatic IQA energies for the interactions between the closest atoms in the ethyne…benzene complex. V_{cl} refers to the exact electrostatic energy obtained by 6D integration, while "FFLUX" refers to this energy approximated by multipole expansion at L'=4 truncation, while the grey histogram represents the absolute energy difference. Note that energy electrostatic energy components are only summed up to L=5.

References

1. R. F. W. Bader, *Atoms in Molecules. A Quantum Theory.*, Oxford Univ. Press, Oxford, Great Britain, 1990.
2. M. Garcia-Revilla, E. Francisco, P. L. A. Popelier and A. Martin Pendas, *ChemPhysChem*, 2013, **14**, 1211.
3. Á. Martín Pendás, E. Francisco, M. A. Blanco and C. Gatti, *Chem. A Eur.J.*, 2007, **13**, 9362.
4. J. C. R. Thacker and P. L. A. Popelier, *J.Phys.Chem.A*, 2018, **122**, 1439−1450.
5. P. L. A. Popelier and D. S. Kosov, *J.Chem.Phys.*, 2001, **114**, 6539-6547.
6. P. L. A. Popelier, L. Joubert and D. S. Kosov, *J.Phys.Chem.A*, 2001, **105**, 8254-8261.
7. B. C. B. Symons and P. L. A. Popelier, *J.Chem.Theory Comp.*, 2022, **18** 5577−5588.
8. Z. E. Hughes, E. Ren, J. Thacker, B. Symons, A. Silva and P.L.A. Popelier, *J.Comput.Chem.*, 2020, **41**, 619.
9. M. A. Vincent, J. L. McDonagh and P. L.A. Popelier, *Chem. Phys. Lett.*, 2016, **662**, 228-234.
10. S. Grimme, J. Antony, S. Ehrlich and H. Krieg, *J.Chem.Phys.*, 2010, **132**, 154104-154122.
11. M. J. Frisch and H. B. S. G. W. Trucks, G. E. Scuseria, M. A. Robb, J. R. Cheeseman, G. Scalmani, V. Barone, G. A. Petersson, H. Nakatsuji, X. Li, M. Caricato, A. V. Marenich, J. Bloino, B. G. Janesko, R. Gomperts, B. Mennucci, H. P. Hratchian, J. V. Ortiz, A. F. Izmaylov, J. L. Sonnenberg, Williams, F. Ding, F. Lipparini, F. Egidi, J. Goings, B. Peng, A. Petrone, T. Henderson, D. Ranasinghe, V. G. Zakrzewski, J. Gao, N. Rega, G. Zheng, W. Liang, M. Hada, M. Ehara, K. Toyota, R. Fukuda, J. Hasegawa, M. Ishida, T. Nakajima, Y. Honda, O. Kitao, H. Nakai, T. Vreven, K. Throssell, J. A. Montgomery Jr., J. E. Peralta, F. Ogliaro, M. J. Bearpark, J. J. Heyd, E. N. Brothers, K. N. Kudin, V. N. Staroverov, T. A. Keith, R. Kobayashi, J. Normand, K. Raghavachari, A. P. Rendell, J. C. Burant, S. S. Iyengar, J. Tomasi, M. Cossi, J. M. Millam, M. Klene, C. Adamo, R. Cammi, J. W. Ochterski, R. L. Martin, K. Morokuma, O. Farkas, J. B. Foresman and D. J. Fox, *GAUSSIAN16*, 2016.
12. J. C. R. Thacker and P. L. A. Popelier, *Theor.Chem.Acc.*, 2017, **136**, 86.
13. I. Alkorta, J. C. R. Thacker and P. L. A. Popelier, *J.Comp.Chem.*, 2018, **39**, 546–556.
14. P. L. A. Popelier, P. I. Maxwell, J. C. R. Thacker and I. Alkorta, *Theor.Chem.Accs.*, 2019, **138:12**, 1-16.
15. Z. M. Sparrow, B. G. Ernst, P. T. Joo, K. U. Lao and R. A. Distasio, *J. Chem. Phys.*, 2021, **155**, 184303.
16. T. A. Keith, *TK Gristmill Software, Overland Park, Kansas, USA*, 2019.
17. P. Maxwell, Á. Martín Pendás and P. L. A. Popelier, *PhysChemChemPhys*, 2016, **18**, 20986-21000.
18. A. D. Becke, *J.Chem.Phys.*, 1993, **98**, 1372-1377.
19. S. Grimme, S. Ehrlich and L. Goerigk, *J.Comput.Chem.*, 2011, **32**, 1456-1465.
20. B. C. B. Symons, M. K. Bane and P. L. A. Popelier, *J.Chem.Theor.Comp.*, 2021, **17**, 7043-7055.
